# Raman spectroscopy reveals age- and sex-related differences in cortical bone from people with osteoarthritis

**DOI:** 10.1038/s41598-020-76337-2

**Published:** 2020-11-10

**Authors:** Michel K. Nieuwoudt, Rayomand Shahlori, Dorit Naot, Rhea Patel, Hannah Holtkamp, Claude Aguergaray, Maureen Watson, David Musson, Cameron Brown, Nicola Dalbeth, Jillian Cornish, M. Cather Simpson

**Affiliations:** 1 The Photon Factory, The University of Auckland, Auckland, 1142 New Zealand; 2 School of Chemical Sciences, The University of Auckland, Auckland, 1142 New Zealand; 3 Faculty of Medical and Health Sciences, The University of Auckland, Auckland, 1142 New Zealand; 4 The MacDiarmid Institute for Advanced Materials and Nanotechnology, Wellington, New Zealand; 5 The Dodd-Walls Centre for Photonic and Quantum Technologies, Dunedin, New Zealand; 6 Department of Chemical and Materials Engineering, The University of Auckland, Auckland, 1142 New Zealand; 7 Medical Engineering Research Faculty, CPME, IHBI, SEF, Queensland University of Technology, Brisbane, Australia; 8 Department of Physics, The University of Auckland, Auckland, 1142 New Zealand

**Keywords:** Physiology, Health care, Optics and photonics

## Abstract

Bone strength in human cortical bone is determined by the composition and structure of both the mineral and collagen matrices and influenced by factors such as age, gender, health, lifestyle and genetic factors. Age-related changes in the bone matrix are known to result in loss of mechanical strength and increased fragility. In this study we show how Raman spectroscopy, with its exquisite sensitivity to the molecular structure of bone, reveals new insights into age- and sex-related differences. Raman analysis of 18 samples of cortical hip bone obtained from people aged between 47–82 years with osteoarthritis (OA) found subtle changes in the lipid and collagen secondary structure, and the carbonate (CO_3_^2−^) and phosphate (PO_4_^3−^) mineral ratios in the bone matrix. Significant differences were observed between older and younger bones, and between older female and older male bones; no significant differences were observed between younger male and female bones. Older female bones presented the lowest mineral to matrix ratios (MMR) and highest CO_3_^2−^/PO_4_^3−^ ratios, and relative to lipid/collagen –CH_2_ deformation modes at 1450 cm^−1^ they had lowest overall mineral content, higher collagen cross linking and lipid content but lower levels of α-helix collagen structures than older male and younger male and female bones. These observations provided further insight on bone composition changes observed in the bone volume fraction (BV/TV) for the older female bones from microCT measurements on the same samples, while tissue mineral density (TMD) measurements had shown no significant differences between the samples.

## Introduction

Our bodies age at different rates that are influenced by our individual genetics and lifestyle. With age, changes in bone architecture and constituents bring about deterioration of both our bone quality, assessed in terms of integrity of the bone internal architecture, and bone quantity, assessed in terms of bone mineral density^1^. Changes in the bone mineral composition with age have been associated with increasing mineral content, hydroxyl content and Ca/P molar ratio, decreasing hydrogen phosphate subsitution and increasing crystal size and perfection^2^, resulting in stiffer and more brittle bones. Changes in the structure of the collagen matrix of cortical bone with age may contribute to increased brittleness and result in loss of toughness. AGE (advanced glycation endproducts) accumulate between the helical parts of the collagen molecules and reducible cross links become non-reducible as the collagen matures, stiffening the collagen matrix^2^.

The structure of cortical bone is complex, consisting of an organic and inorganic composite of hydrated collagen-rich extracellular matrix, intermingled with a rigid, hydrated calcium phosphate mineral lattice substituted with mainly carbonate (CO_3_^2−^) and monohydrogen phosphate (HPO_4_^2−^). Minor amounts of ions may also be present such as fluoride, chloride^3,4^, magnesium, citrate, and other trace elements that depend on what the individual has ingested^5^. The mineral lattice corresponds to a B-type carbonated apatite^6^, with the CO_3_^2−^ ions substituting two different molecular ions, resulting in two different types of substitution: type A (substitution of the OH- ions, creating a vacancy in the lattice) and type B (substitution of the PO_4_^3−^ groups, creating a Ca^2+^ vacancy and a OH^−^ vacancy). The apatite structure in cortical bone is therefore always calcium deficient. The increase in Ca/P ratio with increasing matruation of bone is due to replacement of the HPO_4_^2−^ ions with CO_3_^2−^ ions, while the number of Ca^2+^ ions does not vary^6^. The degree of substitution is also influenced by a number of other factors besides age, such as gender, health, lifestyle and genetics of an individual^7–9^, and is significantly correlated with bone quality and strength^10–14^. Also highly correlated with bone strength is the composition and secondary structure of the collagen matrix (mainly type I collagen with approximately 5% w/w non-collagenous proteins)^4,15,16^. In type I collagen the molecules consist of three chains of amino acid sequences wound together to form triple-helices of elongated fibrils. A special amino acid sequence makes the collagen triple helix particularly stable: every third amino acid is a glycine while most of the remaining amino acids are proline or hydroxyproline. Other amino acids are also present but in smaller amounts. The collagen matrix gives bone its tensile strength so that it carries more deformation and better resists fracture, while the mineral crystals carry more stress^2,4^. The load transfer mechanism between the collagen and hydroxyapatite involves electrostatic interactions in the form of hydrogen bonds and salt bridges^4^.

Sex-related differences in the distribution of mineral (both geometry and morphology) become more pronounced with aging and in the extremely elderly population these differences are believed to contribute to increased incidence of fractures^16–18^. The natural gradient in tissue mineral content due to skeletal growth has also been found to influence the relationship between bone tissue composition and mechanical properties^19–21^. A study measuring nanomechanical properties and Raman spectra of the femoral cortices in growing rats found that tissue modulus, hardness, mineral:matrix ratio (MMR), and PO_4_^3−^/CO_3_^2−^ ratio increased sharply with distance from the periosteum, attaining the properties of intracortical tissue within 4 days of formation^19^. The use of Raman microscopy for assessment of bone composition must therefore take into consideration the positioning of measurement points of the bone cross section surface.

Using micro-computed aided tomography (CT) we can visually assess the bone architecture and measure the bone mineral density as bone volume fraction (BV/TV) and tissue mineral density (TMD). A major challenge in understanding age related changes, particularly in the case of bone, is decoupling the age from the sex of individuals and disease in humans such as osteoporosis. In this study we use bone from patients undergoing hip replacement operations for osteoarthritis; this group of patients was free of osteoporosis. We examine them with both traditional microCT methods and Raman spectroscopy, the latter finding subtle changes to the mineral organisation and in the relationship between collagen and lipid in the bone samples that are differentiated according to age and sex.

## Methods and materials

### Bone samples

All methods in this study were carried out in accordance with relevant guidelines and regulations. Ethical approval was obtained from the New Zealand Northern A Health and Disability Ethics Committee (NTX/05/06/058), and all participants provided written informed consent. 18 cortical bone samples from the femoral neck were collected from patients undergoing total hip replacement surgeries for osteoarthritis and fixed in 70% ethanol in order to preserve the samples. Alcohol storage may cause degreasing and dehydration of the bone samples^22^, however, it would not alter the inorganic matrix^22^. The storage times for these samples ranged between 6 and 12 months, and were in no particular order of age or sex.

The samples were randomized and all stages of the micro CT analysis were blinded. Samples were soaked in saline overnight before being scanned using a Skyscan 1172 micro CT scanner (Bruker micro CT, Aartselaar, Belgium), with X-ray voltage 80 kV, 1 mm aluminium filter; isotropic voxel size 7 µm. After standardised reconstruction using NRecon software the datasets were analysed using CTAn software (Bruker micro CT, Aartselaar, Belgium). Volumes of interest (VOIs) were selected and each dataset was binarised using global thresholding. Parameters were measured including percentage bone volume (BV/TV) and tissue mineral density (TMD). TMD is restricted to the volume of calcified bone and provides information about the material density of the bone itself by comparing X-ray attenuation to that of hydroxyapatite phantoms.

### Raman spectroscopy

**Figure 1:**
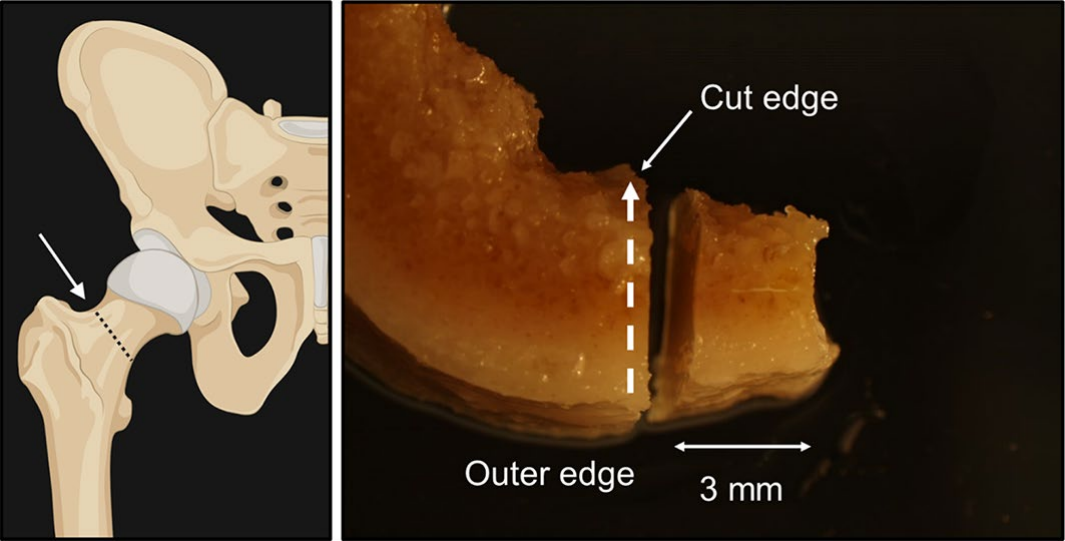
(RHS) Image of cut bone section; spectra were recorded in 500 µm steps, close to the cut edge of the cross section along a line from the periosteal (outer) surface to the endosteal (inner) surface as shown by the direction of the dashed arrow. (LHS, Image created with BioRender.com) showing the area from which the bone sections were sampled.

Raman spectra were recorded of each bone using a Renishaw Raman system 1000 Microprobe, equipped with Leitz microscope with × 50 objective (0.95 N.A.), 50 µm slit width, and TEC cooled CCD. The 785 nm excitation was a Renishaw solid-state diode laser and a Holographic Notch filter (HNF) was used to remove the Rayleigh scattered light. The laser was operated at 100% Power (350 mW at the laser exit) with 60 s accumulation time. A 1200 g/mm grating enabled ~ 6 cm^−1^ spectral resolution. The bone samples were removed from the ethanol solution and left to dry overnight before recording of Raman spectra. For each sample, between 10 and 20 Raman spectra were recorded of 2 µm areas in 500 µm steps along a line from the periosteal (outer) surface to the endosteal (inner) surface as shown by the direction of the dashed arrow in Fig. 1, near the cut edge of the cross section at a distance of about 2 mm from the cut edge of the bone. The figure shows a typical area near the cut edge of the bone sample; the diameter of the cross section varied for each sample according to the sample size. The porous bone surface was not homogeneous, with unevenly sized and distributed crystallites. The number of spectra recorded along the surface varied for each sample and was limited by the length of its cross section. For each point analysed, two spectra were recorded at 60 s integration time and added together to increase the signal to noise ratio.

Bone samples were obtained from participants who were either ≤ 62 years or ≥ 70 years of age. The bone samples were thus classified according to age (older: ≥ 70 years and younger: ≤ 62 years) and gender (male and female). The younger samples ranged from 47–62 years and older samples between 70–82 years. The samples were further grouped into four classes according to both age and sex: younger males (YM) and females (YF) at ≤ 62 years, and older males (OM) and females (OF) at ≥ 70 years. The sample details are summarized in Table 1.

**Table 1:** Sampling information with microCT measurements of TMD and BV/TV.

Sample no	No. spectra averaged	Gender	Age Category	Age	Cort* TMD(g/cm^3^)	Cort^†^ BV/TV (%)
1	10	Female	Young	47	1.061	78.36
2	8	Female	Young	47	1.121	98.64
3	6	Female	Young	58	1.105	94.93
4	4	Female	Young	60	1.069	94.06
5	5	Female	Young	61	1.077	87.82
6	7	Male	Young	51	1.056	85.91
7	12	Male	Young	53	1.093	79.01
8	16	Male	Young	53	1.105	88.15
9	10	Male	Young	58	1.044	92.27
10	9	Male	Young	61	1.141	93.29
11	12	Male	Young	62	1.128	95.36
12	4	Female	Old	70	1.111	70.04
13	5	Female	Old	72	1.138	80.39
14	9	Female	Old	75	1.058	86.61
15	11	Male	Old	74	1.093	81.67
16	13	Male	Old	74	1.100	96.43
17	16	Male	Old	79	1.083	93.08
18	11	Male	Old	80	1.078	89.88

*Tissue mineral density (TMD) of cortical bone.

^**†**^Cortical bone volume fraction (BV/TV) (volume of mineralised bone per unit volume of sample).

The spectra for each sample were baseline corrected using an asymmetric least squares fitting function, with asymmetry factor, p of 0.01 and smoothness, lambda of 5e7, and smoothed using a penalised least squares smoothing (based on Whittaker’s smoothing function)^23,24^ with degree of polynomial d = 2 and smoothness, lambda, of 3. This combination of parameters smoothed the Raman spectra without compromising peak integrity (Figure S1, Supplementary information). The spectra were then normalised to the same height for the δCH_2_ band (C–H deformation) at 1450 cm^−1^, common to proteins and lipids, and commonly selected because it undergoes the least changes^25^. In order to visualize subtle differences between the spectra of the bone samples, the multiple, overlapping vibrational modes known to represent the collagen and mineral components of the bone were resolved using curve resolution. These were the amide I N–C=O stretching region, amide III NH_2_ deformation region, and CO_3_^2−^ and PO_4_ *v*_1_ symmetric stretching regions of the spectra. For this comparison, the spectra recorded along the surface in 500 µm steps for each sample were averaged to mitigate the effect of crystallite orientation heterogeneities in the bone on the spectra, to better represent each sample. The peak resolve function in the OMNIC spectroscopic software was used to resolve the bands, with a mixed Gaussian/Lorentzian fitting algorithm; positions of the band components were selected by a second derivative of the spectral region for each peak region. The FWHM (full width at half maximum) of the PO_4_^3−^ *v*_1_ band resolved at 959 cm^−1^ was used to measure the crystallinity (determined as the inverse of the FWHM)^11,26,27^. Band heights of the 20 most intense bands selected from the averaged spectra were measured and used to create different combinations of band ratios. Using the R and MATLAB software packages, principal component analysis (PCA) was performed on these to select the top 256 showing optimum separation of samples according to age and sex (Table S1, Supplementary information).

The spectra recorded at 500 µm steps from the 18 samples were analysed with PCA in order to explore differences between the samples. In addition, ratios of five of the strongest Raman band intensities for mineral and collagen were selected for two-way analysis of variance (ANOVA) using MATLAB to look for any significant age- and sex-related differences between the samples. Possible effects of tissue age on these ratios were also investigated for all samples using one-way ANOVA on the spectral ratios, from the spectra recorded at the 500 µm step positions along the cross sections from the periosteal to endosteal surfaces.

## Results

**Figure 2:**
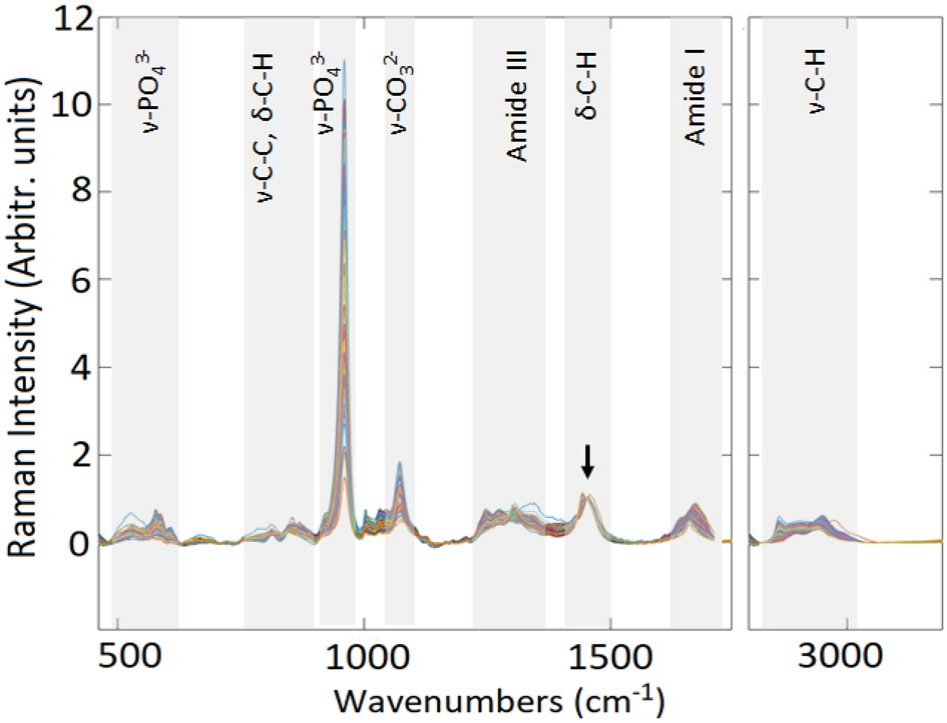
Raman spectra in overlaid format, of spectra recorded in 500 µm steps along the cross sections of the 18 bone samples, normalized to the 1450 cm^−1^ collagen –CH_2_ deformation band, indicated by the arrow. The relative intensities of both the mineral and other collagen matrix bands of the different samples and sampling positions differ relative to this band.

The Raman spectra recorded of all 18 samples are shown in overlaid format in Fig. 2. The spectra have been baseline corrected, smoothed and normalized to the collagen –CH_2_ deformation mode at 1450 cm^−1^, with the region between 1780 and 2800 cm^−1^ excluded as it has no relevant spectral information for these samples. Six spectra were excluded as outliers, having reduced Q residuals and reduced Hotelling T^2^ > 3.

### PCA and ANOVA of spectra recorded of the cortical bone samples

**Figure 3:**
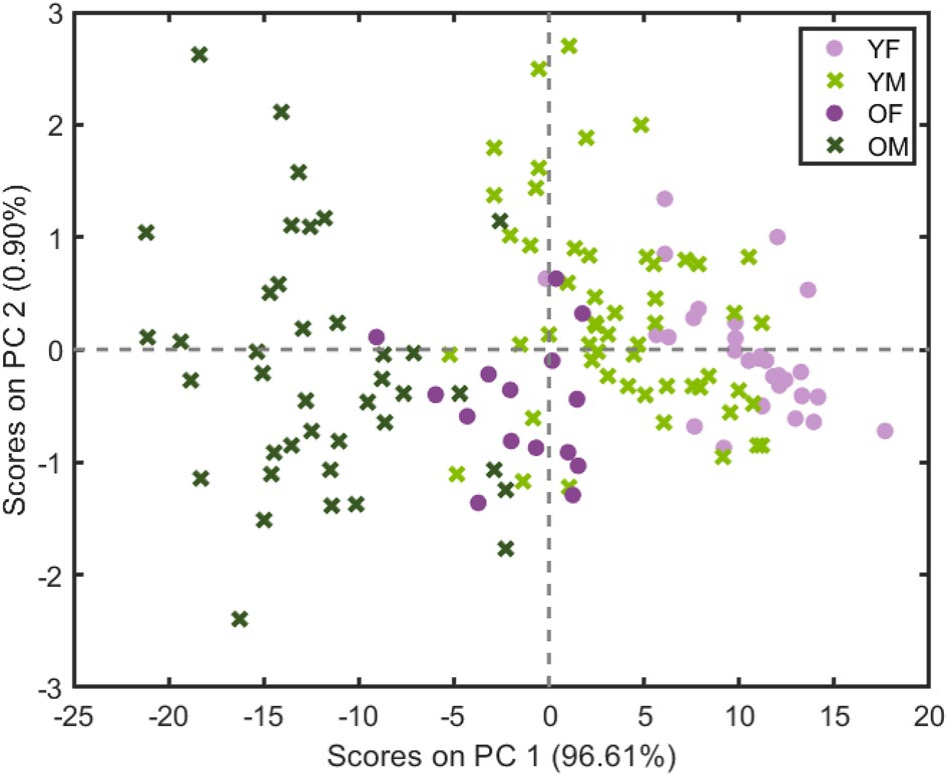
Scores for PC1 and PC2 from PCA of the spectra recorded at different positions of all 18 samples, accounting for 97.51% of the variance in the set. Clustering of the samples is apparent along PC1, particularly between older and younger samples with some grouping for the male and females within each age group. The loadings for PC1 and PC2 are given in Fig. 4, describing the spectral variables contributing to these groupings.

PCA was performed on the spectra shown above in Fig. 2. The scores are plotted in Fig. 3 for the first two principal components (PC’s), which explained 97.5% of the variance in the samples. Clustering of the samples according to age is apparent along PC1 with older and younger samples grouped along negative and positive loadings, respectively. The older males (OM) and younger females (YF) samples are clearly separated, with some of the spectra from older female samples (OF) showing overlap with some of the younger male (YM) samples and also OM samples, while YM and YF show more extensive overlap.

**Figure 4:**
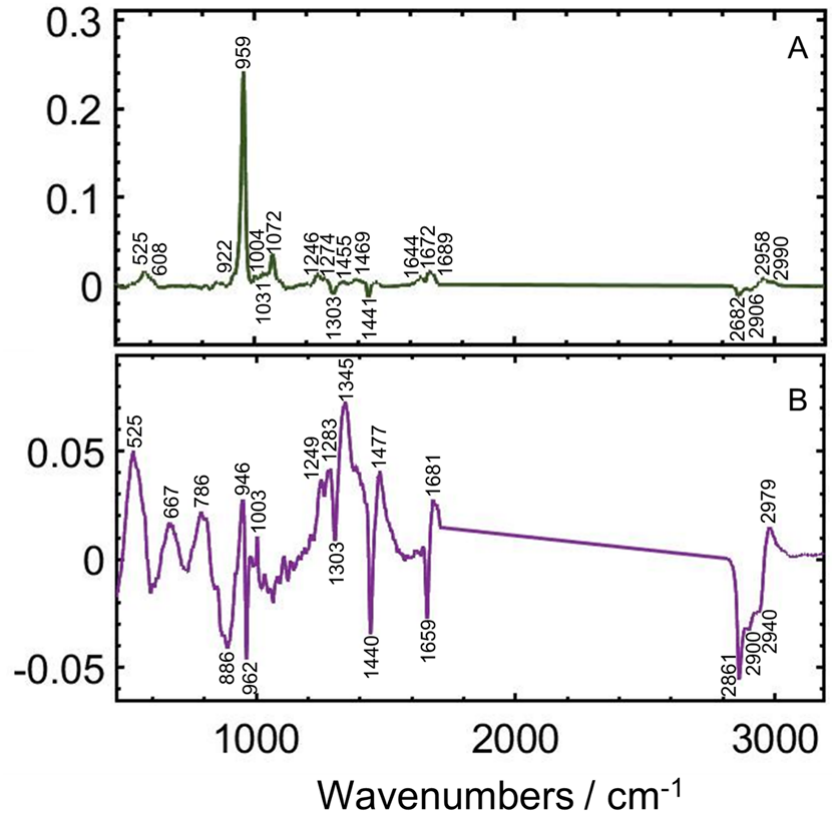
(**A**) Loadings for PC1 and (**B**) for PC2, from PCA of 158 spectra recorded of the 18 cortical bone samples. The positive loadings in (**A**) are associated with younger males and females, and the negative loadings are associated with the older males and females. (**B**) Shows the positive and negative loadings which differentiate samples within each group.

The younger samples are associated with greater relative intensities of bands indicated by the positive loadings for PC1 in Fig. 4A, and the older samples with greater negative loadings. Within each age and sex group samples are spread along PC2, with negative loadings representing A-substituted CO_3_^2−^ and HPO_3_^2−^ (indicated by the higher loading for 946 cm^−1^ and 1003 cm^−1^ bands, respectively) and a sharp negative loading for PO_4_^3−^ at 962 cm^−1^ which may be either lower crystallinity manifesting a broader 959 cm^−1^ peak, or a shift in the PO_4_^3−^. Most of the OF are associated with negative PC2 loadings. No grouping of the scores within each age/sex group according to measurement position was apparent along PC2.

**Figure 5:**
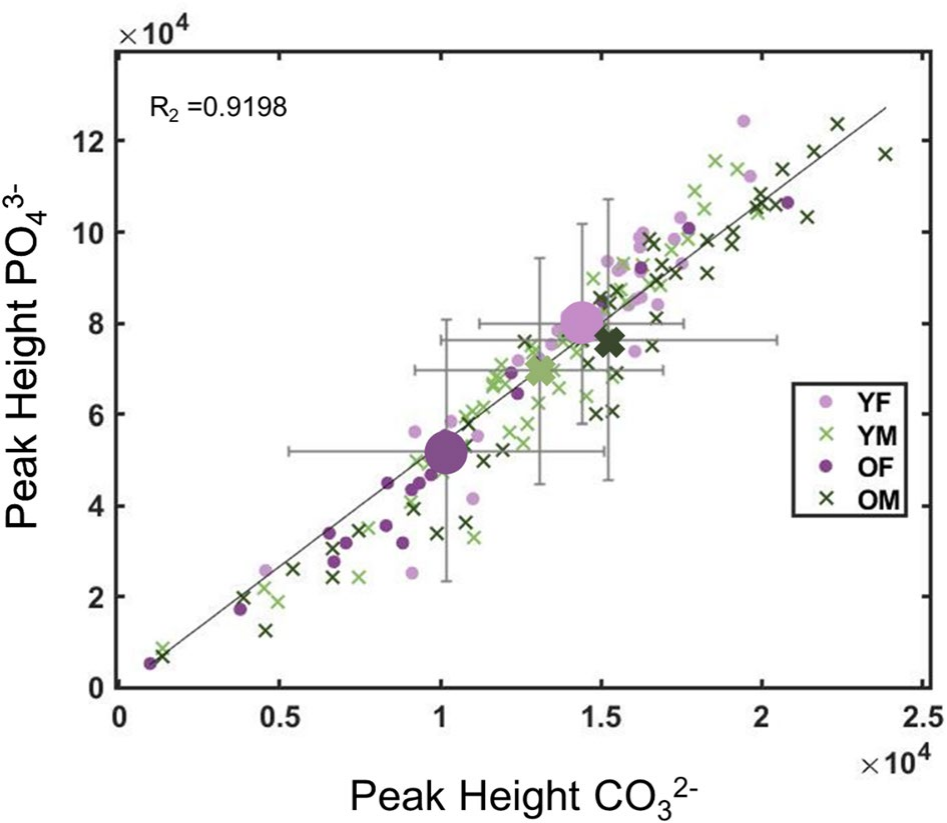
Relationship between the B-CO_3_^2−^ and PO_4_^3−^ band intensities of all 158 spectra recorded at the different measurement positions, for YF, OF, YM and OM. The large symbols represent the mean values for each group.

Figure 5 shows a 2-D scatter plot of the band intensities for all 158 spectra of the PO_4_^3−^ stretching mode at 959 cm^−1^ and B-substituted CO_3_^2−^ at 1072 cm^−1^; the average band intensities for each of OF, OM, YF and YM are represented by the larger symbols. Individual measurement positions of all four groups are spread across the range, however, on average the older females show lowest PO_4_^3−^ and CO_3_^2−^ intensities.

### ANOVA of selected Band ratios and TMD, BV/TV

A two-way ANOVA was performed on five selected ratios of peak heights of the spectra, (CO_3_^2−^/PO_4_^3−^, CO_3_^2−^/Amide 1, PO_4_^3−^/Amide 1, 1441/1454 cm^−1^ and 1662/1690 cm^−1^), as well as the TMD and BV/TV measurements, to investigate whether there were significant differences between age, sex and any interactions between the four age/sex groups. The p-Values are given in Table 2.

**Table 2:** Two-way ANOVA results for p-values for significance of the effects of the five selected band ratios, TMD and BV/TV measurements on age, sex or age/sex interaction.

Band ratios	Age (p-values)	Sex (p-values)	Age/sex interaction (p-values)
CO_3_^2−^/PO_4_^3−^ cm^−1^	0.0038***	0.4100	0.4100
1441/1454 cm^−1^	0.0002***	0.8753	0.4504
PO_4_^3−^/Amide 1	0.0001***	0.7335	0.0416**
CO_3_^2−^/Amide 1	0.0028***	0.7614	0.0678*
1660/1690 cm^−1^	0.0186**	0.3543	0.5748
BV/TV	0.0274**	0.0444**	0.0072***
TMD	0.7031	0.6674	0.2706

Significant differences occurring at three confidence levels are marked with asterisks as p < 0.01***, p < 0.05** and p < 0.1*.

The average values of these ratios for each of the four groups are plotted in Figure S4 (Supplementary Information). Peak intensities for the PO_4_^3−^ (959 cm^−1^), B-CO_3_^2−^ (1072 cm^−1^), amide I (1662 cm^−1^) bands, relative to the 1450 cm^−1^ –CH_2_ band intensity, are given in Figure S5.

A one way ANOVA was also performed on these ratios for the different measurement positions, at 500 µm intervals starting from the periosteal to endosteal surface, to check for differences resulting from tissue age. ANOVA was performed on the whole sample set and also within each age/sex group. The mean values of the band ratios for the whole set at different measurement positions are plotted in Figure S3, Supplementary information. No significant differences in any of the five ratios were observed for measurement position. For the ANOVA of the measurement positions within each age/sex group, significant differences were found in some band ratios due to measurement position for the YM group only: for the PO_4_^3−^/Amide 1(p = 0.0175), 1660/1690 (p = 0.0064) and 1441/1451 cm^−1^ ratios (p = 0.0001) (Figure S6, Supplementary Information).

### Band resolution of averaged spectra of the 18 cortical bone samples

**Figure 6:**
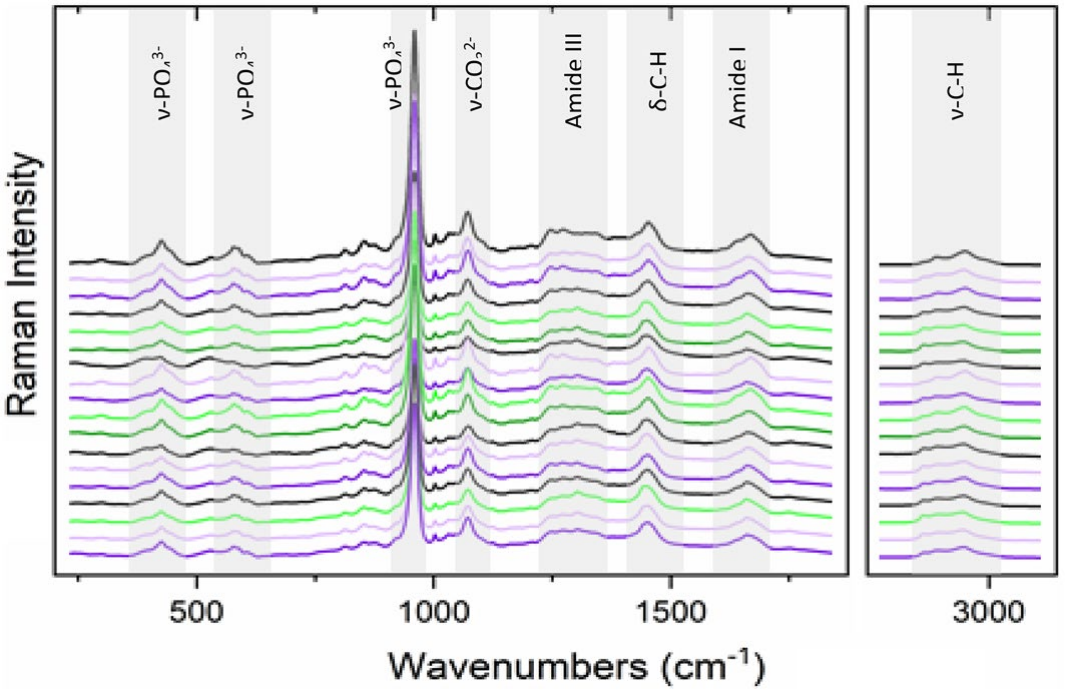
Raman spectra of the averaged 18 cortical bone samples in offset format, from youngest at the top to oldest at the bottom, after baseline correction and scaling to the same band height for the C–H deformation mode of the –CH_2_ groups at 1450 cm^−1^, common to proteins and lipids. The vibrational assignments of the different band positions are given in Table S2 (Supplementary information).

In order to more closely examine differences in the collagen and mineral bands between the samples, the Raman spectra were averaged for each of the 18 samples; these are plotted in Fig. 6 in offset format for clarity (after baseline correction and scaling to the same height for the band representing CH_2_ deformations, at 1450 cm^−1^). The strongest bands are labelled and the band assignments are given in Table S2 (Supplementary Information), along with others reported in the literature. Differences between the samples are seen particularly in the relative band intensities of the PO_4_^3−^ bands (~ 959 and 500–600 cm^−1^). Other differences occur in the complex amide I vibrational mode (1620–1700 cm^−1^), due to C=O stretching vibration of the N–C=O functional groups in the amide backbone), and amide III mode (1200–1300 cm^−1^, representing the NH_2_ deformations)^27–30^. Differences also occur in the CH_2_ and CH_3_ deformation modes (δ(C–H) ~ 1440–1460 cm^−1^) and the CH_2_ and CH_3_ stretching modes (ν(C-H) ~ 2800–3100 cm^−1^).

**Figure 7:**
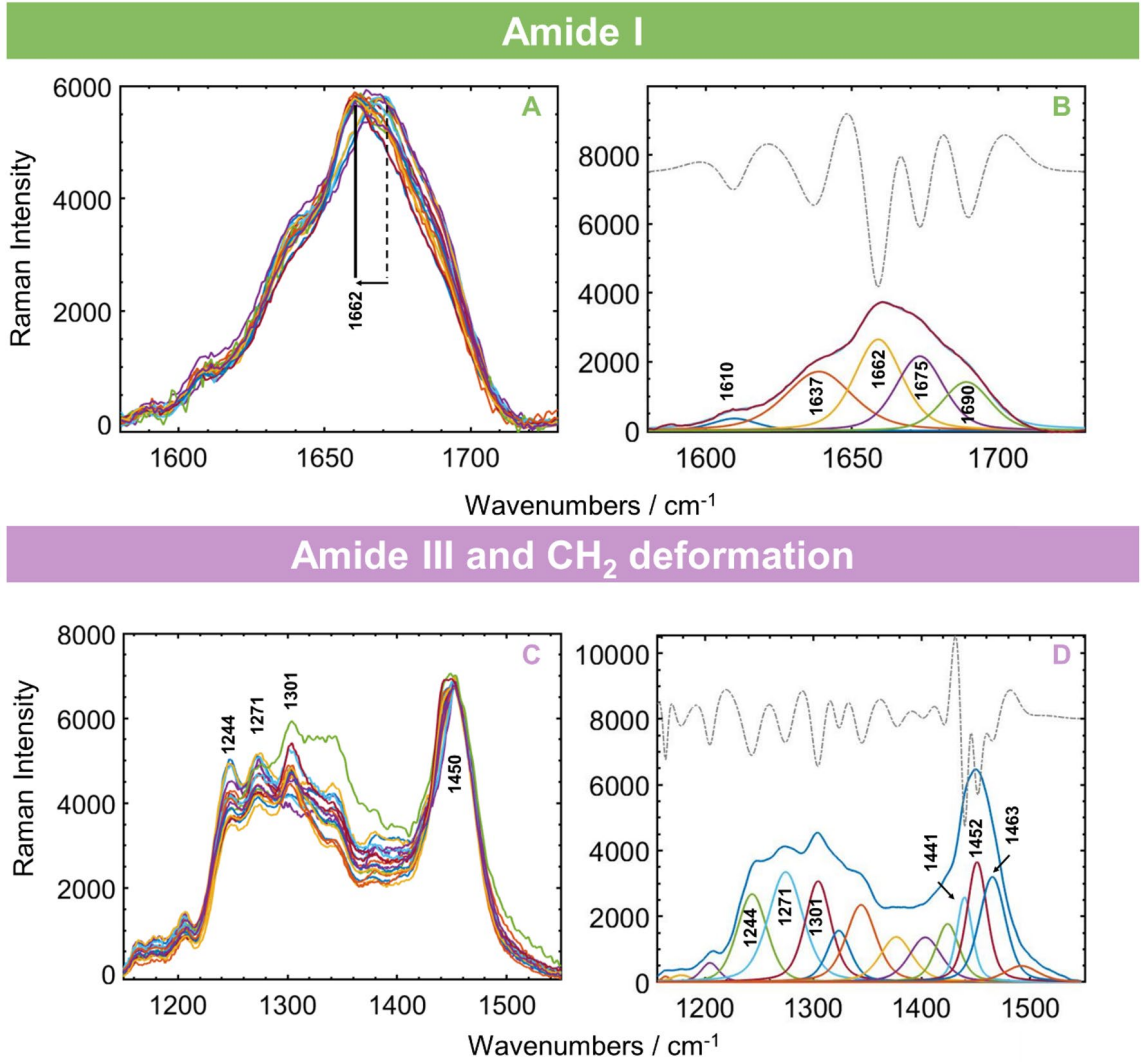
(**A**) Overlapped amide I band region for all 18 cortical bone samples, showing apparent shifts in the 1662 cm^−1^ band. (**B**) Band resolution (shown for one sample) of individual components arrows mark the 1660 and 1690 cm^−1^ components which undergo changes in relative intensities with age. Also plotted are second derivative spectra used to guide number of components fitted. (**C**) Amide III band region with adjacent C–H deformation region around 1450 cm^−1^, (**D**) resolved components; arrows mark both C–H deformation components between 1440–1470 cm^−1^ and amide III components 1271 and 1244 cm^−1^ which undergo changes in relative intensities.

**Figure 8:**
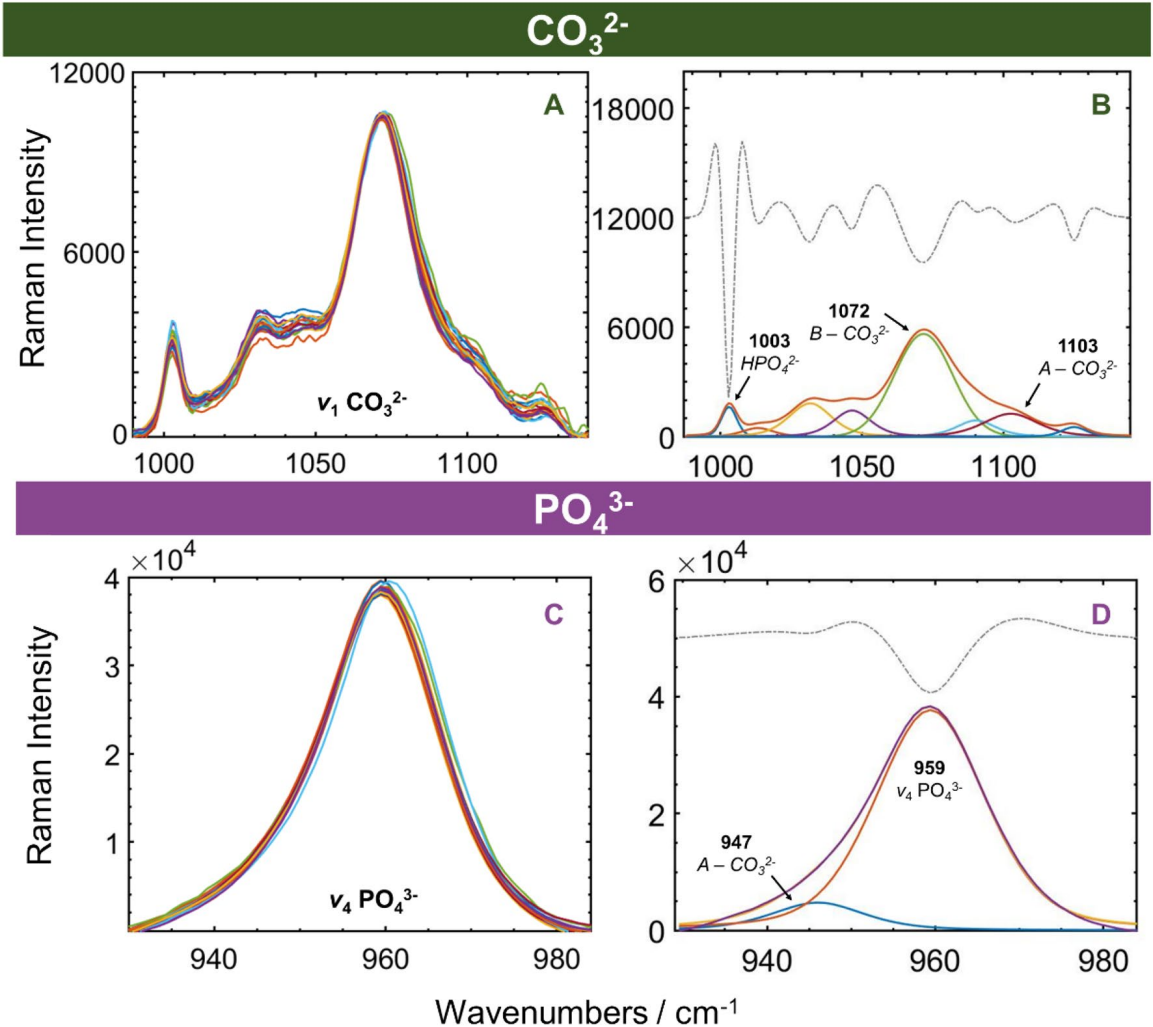
Overlapped spectra of the 18 samples, showing differences in relative intensities for the different mineral band components. Also plotted are second derivative spectra used to guide number of components fitted. All spectra were scaled to the same height for the 1450 cm^−1^ CH_2_ deformation mode shown in (**C**) before truncating to the selected regions for band resolution. (**A**) CO_3_^2−^ ν_1_ stretch modes for B-substituted CO_3_^2−^ and (**B**) shows the resolved bands for this region. (**C**) PO_4_^3−^ symmetric stretch region with (**D**) the resolved PO_4_^3−^ and the A-substituted CO_3_^2−^ mode at 947 cm^−1^.

Many of the phosphate and monohydrogen phosphate modes overlap extensively with those of the carbonate^31^ and collagen^30^ vibrational modes, making it difficult to accurately assign the individual bands. Changes in relative amounts of random coil and helix structures have been measured from resolved band component intensities for the amide III band at 1243 cm^−1^ and 1273 cm^−1^, respectively^11,25,26,29,30,32^, and from components in the amide I band (1700–1600 cm^−1^)^33^. The averaged amide I spectral region for each of the 18 samples are shown in overlaid format in Fig. 7A, and those for amide III in Fig. 8A. We applied curve resolution to resolve the contributing components of the amide I band (Fig. 7B), as well as the amide III bands (Fig. 7D) which overlaps extensively with neighbouring C–H deformations (1300–1500 cm^−1^). Curve resolution was also applied to measure the components of the CO_3_^2−^ symmetric stretch band centered at 1072 cm^−1^ (Fig. 8B) and in the PO_4_^3−^ symmetric stretch mode at 959 cm^−1^ (Fig. 8D). The second derivative spectra that were used to select the fitted band positions are included in the figures for each set of spectra.

Five components were required for optimal resolution of the amide I band, as shown for one of the samples in Fig. 7A. The strongest band components at 1662 and 1675 cm^−1^ represent α-helices^21,25,27^, while the bands at 1634 and 1690 cm^−1^ represent random coiled structures^11,30,34^. The 1660 cm^−1^ band has also been assigned to pyridinoline (non-reducible) cross-linking, amide I C=C and C=O stretching in collagen, CH_2_/CH_3_ stretching and deformation, NH_2_ stretching and deformation of lipids and collagen^9,20,24,26,35^.

The red shift (to lower frequencies) indicated by arrows in the amide I maximum, with increasing age, appears to be caused by an increase in the ratio of the band component at 1660 cm^−1^ relative to that at 1690 cm^−1^ (1683 cm^−1^)^36^. The ratio of α-helices to random coiled structures thus appear to increase with age. This ratio has been referred to as the collagen cross link ratio, but also as matrix mineralization or matrix maturity ratio and has been used to measure the extent of cross linking and maturity of the collagen^11,36–38^. Similar increases in this ratio with age have also been observed in mural, chick and bovine bones^33,36,39^.

Measurement of the amide III components between ~ 1200–1320 cm^−1^ is complicated by the extent of overlap of lipid and collagen –CH_2_ deformation modes between 1300–1500 cm^−1^ in the complex envelope which covers the range 1150–1550 cm^−1^ (Fig. 7C). Second derivative treatment determined that 15 components were needed to adequately resolve the envelope (Fig. 7C,D). The samples showed clear differences in relative intensities of the amide III band components at 1301 cm^−1^ (attributed to lipids)^40,41^, 1246 and 1247 cm^−1^, marked in Fig. 7D. The 1301 cm^−1^ component has been assigned to deformations of phospholipids and of –CH_3_ and –CH_2_ groups in collagen and lipids^3,29,30^. The 1246 cm^−1^ component arises from random coil structures and the 1273 cm^−1^ component is assigned to α-helix structures. Differences in relative amounts of these components observed for older and younger samples are another indication of differences in collagen secondary structures between the samples.

Band resolution of the symmetric stretching vibrational modes for CO_3_^2−^ and PO_4_^3−^ symmetric stretch mode in Fig. 8A and C, respectively, are shown for one of the samples in Fig. 8B and D, respectively. The fitted bands revealed differences between the samples in the relative intensities of the A-substituted CO_3_^2−^ components at 947 cm^−1^ and 1103 cm^−1^, in the B-substituted CO_3_^2−^ band at 1072 cm^−1^, and in the 1003 cm^−1^ band attributed to monohydrogen phosphate, HPO_4_^2−^^3^. It should be noted that while the 1003 cm^−1^ is also characteristic for phenylalanine, this is present in much lower amounts in the collagen type I in bone than in type IV collagen^28^.

Figure 8D shows the resolution of the A-CO_3_^2−^ component at 947 cm^−1^ that overlaps with the 959 cm^−1^ symmetric PO_4_^3−^ stretching mode^11,26^; the asymmetry it affords to the PO_4_^3−^ band is seen in Fig. 8C. The crystallinity was measured as the inverse of the FWHM of the resolved PO_4_^3−^ component at 959 cm^−1^. A third CO_3_^2−^ has been identified in FTIR studies appearing at 866 cm^−1^ and attributed to a labile species related to early stage of apatite crystal formation^42^. The 866 cm^−1^ band is possibly an out-of-plane deformation red-shifted from that of 874 cm^−1^ for lattice-bound CO_3_^2−^ species; the labile CO_3_^2−^ species could possibly also be detected in the Raman spectra as a symmetric stretch mode near 1072 cm^−1^, and may be represented by one of the fitted bands for CO_3_^2−^ in Fig. 8B.

A correlation heat map between the resolved band components and physical measurements TMD and BV/TV (Figure S7, Supplementary information) shows links between unique resolved Raman band components and physical properties over all 18 samples. Age and sex showed some correlation with the different mineral and collagen components and crystallinity. There was no correlation of age with TMD and BV/TV, with only a weak positive correlation of BV/TV with sex. Comparison of the BV/TV and porosity (= 100-BV/TV) between the samples showed that the slight positive correlation is due to the older females having higher porosity than the other age groups (Figure S8, Supplementary information).

**Figure 9:**
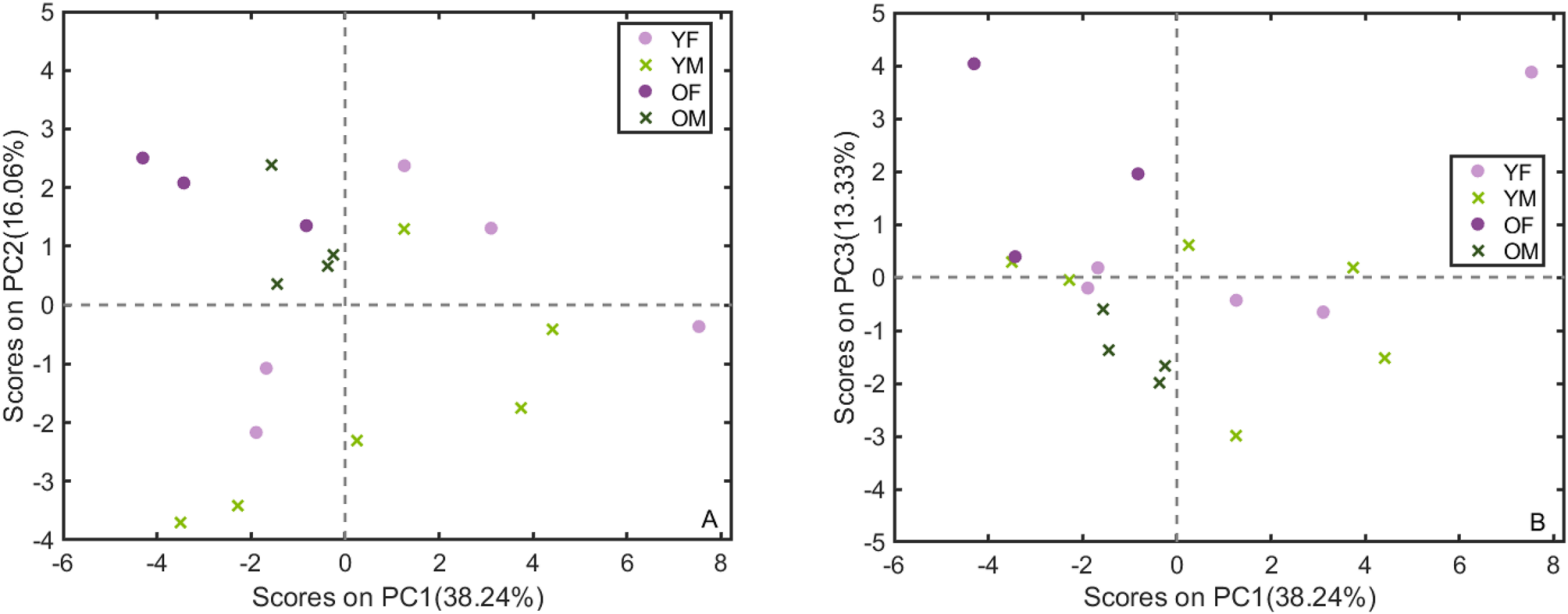
(**A**) Scores plotted for PC’s 1 and 2 from PCA of band areas, band ratios from averaged spectra and physical parameters of the 18 cortical bone samples. (**B**) PC’s 1 and 3 from PCA of band areas, band ratios from averaged spectra and physical parameters of the 18 cortical bone samples.

**Figure 10:**
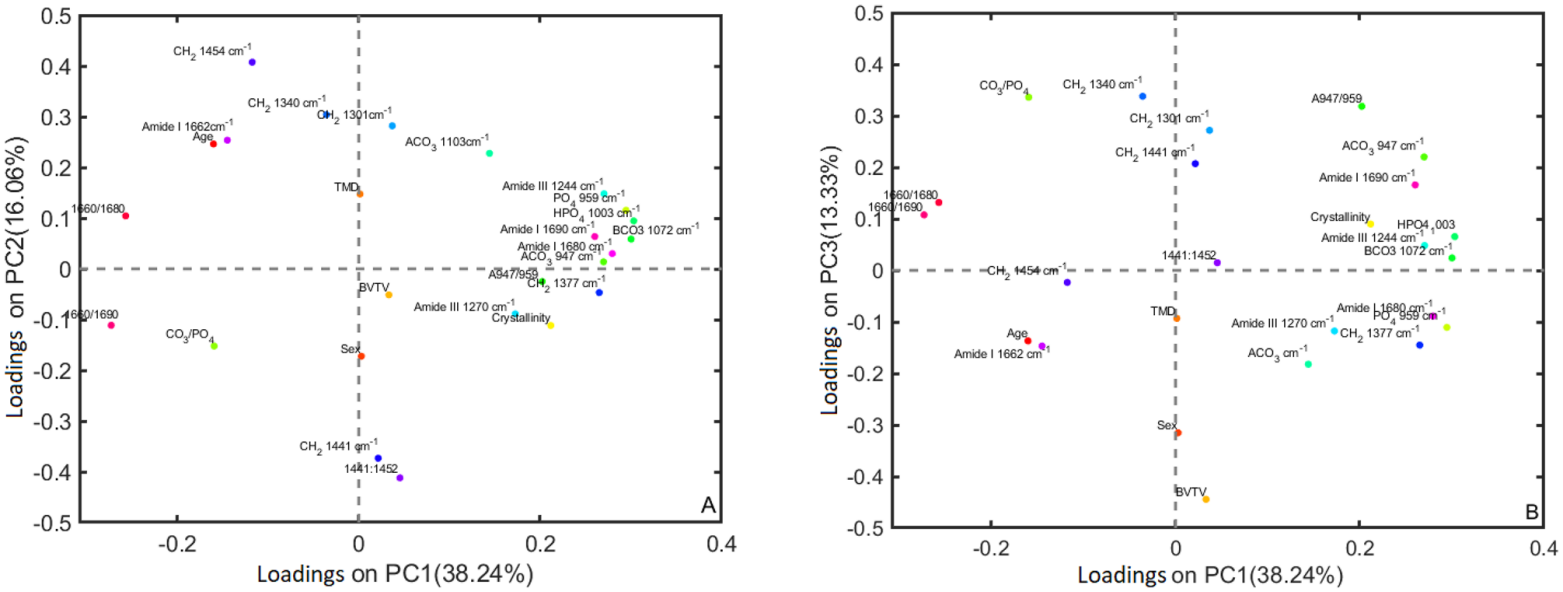
(**A**) Loadings plotted for PC’s 1 and 2 from PCA of band areas, band ratios from averaged spectra and physical parameters of the 18 cortical bone samples. (**B**) PC’s 1 and 3 from PCA of band areas, band ratios from averaged spectra and physical parameters of the 18 cortical bone samples.

In order to examine more closely the relationship in these correlations, a PCA was performed on peak areas and selected peak area ratios of the main resolved Raman band component areas of the averaged spectra for the 18 samples (amide I, amide III, C-H deformation mode, PO_4_^3−^ and CO_3_^2−^ stretching modes) as well as crystallinity, TMD and BVTV values. Six PC’s were required to explain 90% of the variance. The scores for PC’s 1–3 are given in Fig. 9A and B), and the corresponding loadings in Fig. 10A and B. The scores and loadings for PCs 4 to 6 are given in Figures S9 (A) to (F), (Supplementary information).

## Discussion

The first two PC’s of the PCA of the spectra explain 97% of the variance in the data (scores and loadings are given in Figs. 3 and 4, respectively). Separate grouping along PC1 scores is apparent according to age, with the younger samples having greater band intensities corresponding with positive loadings along PC1 in Fig. 4A. These include bands at 578 and 608 cm^−1^ (PO_4_^3−^ deformations)^26–28,31^, 922 cm^−1^ (deformation modes for C=O, C–C and C–H groups in proline, hydroxyproline and collagen)^25^, 959 cm^−1^ (PO_4_^3−^ symmetric stretch)^26,27,30^, 1004 cm^−1^ (HPO_4_^2−^ symmetric stretch)^3,30,31^, 1031 cm^−1^ (PO_4_^3−^ symmetric stretch and proline)^30^, 1072 cm^−1^ (CO_3_^2−^ symmetric stretch)^3,26,27,30,31^, 1246 and 1274 cm^−1^ (random coil and α-helix collagen structures, respectively)^11,27,29,30^, 1455 and 1469 cm^−1^ (protein δ-C–H modes)^25,29,30,43^, 1672 cm^−1^ (Amide 1 α-helix collagen structures)^30,43^, 1644 and 1690 cm^−1^ (Amide 1 random coil structures)^11,25,44^, 2958 cm^−1^ (protein and lipid C–H stretching)^30,33,37^, and 2990 cm^−1^ (collagen C–H stretching)^27,30^.

### Lipid and collagen components

The older samples show greater intensities in bands associated with negative PC1 loadings, which represent lipids and some secondary collagen structures: the 1303 cm^−1^ (due to δ(=CH) phospholipids, δ_as_(CH_3_) and δ(CH_2_) of collagen and lipids), 1441 cm^−1^ (lipid and protein δ-C–H bands and NH_2_ stretching and deformation in collagen)^25,29,30,43^ and 2862 and 2906 cm^−1^ modes (lipid ν(C–H) stretching)^25,29,30,43^. Although the C–H stretch modes between 2800–2960 cm^−1^ represent both proteins and lipids in the collagen matrix, bands occuring at 2852, 2856–2862, 2882–2886 cm^−1^ are attributed mainly to lipids^26,29,35,45^.

Bearing in mind that all spectra are normalized to the same height for the –CH_2_ deformation 1450 cm^−1^ band, separation of scores according to the bands described above show that relative to this mode, the younger bone samples generally have greater levels of B-CO_3_^2−^, PO_4_^3−^ and collagen amide structures than older bone. The older samples, on the other hand show higher amounts of lipids and some secondary collagen structures. Clear separation of OM from YF samples is based on these compositional differences. The OF samples overlap with the YM samples that were the two oldest of the YM samples (10 and 11, aged 61 and 62). YM and YF bones also overlap to some extent, as do some of the OM and OF samples, however, some degree of clustering of samples along PC1 is evident within each age group according to sex. The larger separation observed between older male and female samples compared with younger male and female samples suggests that in males, change in mineral composition and lipid content occur to a greater extent with ageing. In females, the lipid content is increased the older female samples but the extent of increase with age is lower than in the older male samples. Bone marrow is 88–95% triglyceride^41^ but lipids are also present in bone-mineralized tissue and in the bone cells where they are loosely-bound/easily extractable, while the mineralized collagen matrix has more tightly associated lipid complexes together with proteins and other minerals^7,9,46,47^. Lipid content is increased during times of vascular invasion^41^, however, this may not be linked with the older samples.

The scores and loading plots of PCA of fitted components of averaged in Fig. 9A and B and the correlation heat map in Figure S7 (Supplementary Information) show positive correlation between older samples and the lipid 1301 and 1340 cm^−1^ and 1454 cm^−1^ –CH_2_ modes. Fitting of the bands show the 1441 cm^−1^ to be very low in the older bone samples with the 1454 cm^−1^ band dominating. The –CH_2_ 1377 cm^−1^ component, however, appears to be of greater intensity in younger samples, indicating changes in the collagen matrix secondary structures with ageing. The PC 1 and 3 scores and loadings of the band area ratios of averaged spectra (Figs. 9B and 10B) show that OF samples have greater intensity for the 1441 cm^−1^, the 1301 and 1344 cm^−1^ band components than the older males and most of the younger samples. These observations suggest that OF differ from OM in lipid and collagen secondary structure. The seeming discrepancy in the levels of 1441 and 1454 cm^−1^ observed in older and younger samples, between the spectral PCA loadings in Fig. 4 and the fitted band ratio PCA loadings in Figs. 9B and 10B, show the need to resolve these highly overlapped bands for a more accurate measure of the 1441 and 1454 cm^−1^ components, as opposed to measuring the shoulder height in the spectrum.

Tissue age has been shown to affect the lipid content in bone^20^, and also the tissue modulus, hardness, MMR, and CO_3_^2−^/PO_4_^3−^ ratio^19^. However, these studies were on rat bone and human trabecular bone which are different from cortical bone, and the findings in these studies were based on measurement steps of 20–30 µm from the surfaces^19–21^. The p-values and average values from one-way ANOVA on all spectra recorded at 500 µm steps in this study (Figure S3, Supplementary Information) show no significant effect of measurement position on band ratios. Two-way ANOVA of measurement positions within each age/sex group did find YM showing significant differences for some ratios with measurement position (Figure S6, Supplementary Information), however, this did not appear to affect the distribution of spectral PCA scores in Fig. 3. We could not find direct evidence or references to suggest which aspect (periosteal vs. endosteal) of human cortical bone is older; this could be further investigated.

Also strongly positively correlated with age is the collagen cross linking ratio (1660/1690 and 1660/1680)^11,36,42^ (Figs. 9A,B, 10A,B) and Figure S7, Supplementary Information). The 1660/1690 ratio has also referred to as the collagen maturity ratio or the MM (matrix mineralization) ratio^11^. Both 1660/1690 cm^−1^ and 1660/1680 cm^−1^ ratios have high negative PC1 loadings in Fig. 9B, associated with all older samples. An increase in these ratios with age manifests as a red shift of the amide I band maximum; the 1660/1680 cm^−1^ ratio in particular has positive PC2 and negative PC1 loadings which specifically separates all the older samples from all younger bone samples (Fig. 9A). The OF are further separated from the OM along PC3 with higher levels of both 1660/1690 cm^−1^ and 1660/1680 cm^−1^. The increase in cross linking ratio with age corresponds with the increased lipid modes observed and changes in the secondary structure of the collagen matrix^16,48^; it also is linked with changes in the crystallinity and mineralization of the matrix^3,7,48,49^. This increase in cross linking ratio in older samples is also associated with their lower amide III α-helix band components (1270 cm^−1^) and higher random coiled structures (1244 cm^−1^) (Fig. 9A and B), which correlate with gradual deterioration of the mechanical properties of bone. The 1660 band has been assigned to α-helix structures^21^, pyridinoline (non-reducible) cross-linking, amide I C=C and C=O stretching in collagen, CH_2_/CH_3_ stretching and deformation, NH_2_ stretching and deformation of lipids and collagen^9,20,24,26,35^.

For individual alpha helices, which are the fundamental structures in the right-handed triple helix, this mode occurs at 1650 cm^−1 ^^50,51^, however, the unique arrangement of amino acid sequences and hydrogen bonds between them result in the shift observed here from 1650 cm^−1^ for the ‘free” alpha-helices, to a higher energy at 1660 cm^−1^ for those in the triple helices.

Changes in the cross linking ratio have been attributed to changes in the pyridinoline and dehydrodihydroxy-lysinonorleucine (reducible) cross-linking at 1690 cm^−1 ^^11,33,36,38,39,52,53^. However, another study suggested the increase in 1660/1690 ratio reflecting changes in the secondary structure of collagen was more likely linked to dehydration of the mineral phase^44,54^. The increased collagen cross linking in the OF samples is also associated with an increase in B-CO_3_^2−^/PO_4_^3−^ ratio (Figs. 9B and 10B), and is also positively correlated with B-CO_3_^2−^/PO_4_^3−^ (Figure S7, Supplementary Information), supporting suggestions that these amide I band components are associated with mineralized tissue type I collagen cross-links^33^. OF are also associated with the lowest levels of α-helix structures (1660 cm^−1^ amide 1 and 1271 cm^−1^ amide III) (Figs. 9B and 10B).

### PO_4_^3−^ and CO_3_^2−^ mineral components

A two-way ANOVA of the band ratios of PO_4_^3−^ and CO_3_^2−^ with amide 1 (MMR, 959/1660 and 1072/1660 cm^−1^), CO_3_^2−^/PO_4_^3−^, BV/TV as well as the 1441/1454 and 1660/1690 ratios, showed significant differences between older and younger samples, and significant age/sex interaction for the PO_4_^3−^/amide 1 ratio and BV/TV (Table 2). PCA of the spectra grouped the scores of older and younger samples separately according to PO_4_^3−^/B-CO_3_^2−^ (Figs. 3 and 4).

The band intensity for PO_4_^3−^ at 959 cm^−1^ is plotted vs that for B-CO_3_^2−^ at 1072 cm^−1^ for all spectra in Fig. 5, and shows spreading of all age/sex groups over a wide range; it also shows a linear trend. Overall, OF have lower intensities for B-CO_3_^2−^ and PO_4_^3−^ (this is relative to the 1450 cm^−1^ band); OF and OM have lower B-CO_3_^2−^/PO_4_^3−^ than the younger sample groups (Fig. 9A,B) and Figures S4 and S9(A), Supplementary Information), but OF have higher B-CO_3_^2−^/PO_4_^3−^ ratios than OM (Figs. 9B and 10B). The A-CO_3_^2−^ fitted band areas for 947 cm^−1^ and 1103 cm^−1^ are also present in smaller amounts for older bone samples (Figs. 9A and 10A). However, the PC1 and PC3 scores further separate the OF from the OM, with OM having lower intensities for the A-CO_3_^2−^ bands than the OF (Figs. 9B and 10B). The YF and YM mostly have higher levels of A-CO_3_^2−^_._

The lower levels of B-CO_3_^2−^ and PO_4_^3−^ in OF correlates with the higher porosity observed for this group (Figure S8, Supplementary information) from BV/TV measurements, and the ANOVA p-values in Table 2 show significant effects of both age and sex of BV/TV and the age and age/sex interaction for the mineral to amide 1 band ratios. The porosity, which is the vascular canal network that enables fluid flow throughout cortical bone, was the only microCT parameter that flagged the age-related differences for the older female group. The TMD measurement which quantifies the mean mineralization of the bone tissue, excluding the pores^55^, did not show meaningful differences between the groups, however, PCA did show YM as a group are associated with higher TMD levels (Figures S9(C) and (D), Supplementary Information).

Increasing mineralization of bone with age is accompanied by increased porosity and hypermineralization, and have been associated with the decrease in its mechanical properties^16,17,56^. With increased ageing, CO_3_^2−^ substitution of the PO_4_^3−^ in the hydroxyapatite matrix has been observed, creating internal strains in the bone matrix which negatively affect fracture resistance^43,49^. Studies have shown that increasing cortical bone stiffness, bending modulus, yield displacement, yield, and yield strain are correlated with age^2,9,15,57^. The results obtained here support the increased CO_3_^2−^/PO_4_^3−^ with age and particularly in OF, and also show that OF on average show lowest levels of PO_4_^3−^ and CO_3_^2−^, however, the OM had lower A-CO_3_^2−^ than the OF. The results also show that the OF group show the lowest levels of α-helix structures (1660 cm^−1^ amide 1 and 1271 cm^−1^ amide III) (Figs. 9B and 10B). The combination of lower levels of PO_4_^3−^ and CO_3_^2−^, high CO_3_^2−^/PO_4_^3−^ ratios and lower levels of α-helix structures in OF would contribute to their low fracture resistance. The linear relationship in the plot of PO_4_^3−^ vs B-CO_3_^2−^ band intensities reflects the differing rates of bone mineralization for individuals, and could enable comparison of the level of type B-CO_3_^2−^ substitution. Further work is underway to extracting compositional and structural information from SORS spectra of bone, measured using a spatially offset Raman spectroscopy (SORS) probe that can be applied to the skin but is still able to measure at suitable depth to generate a strong Raman response unique to bone. Insight from the molecular signature contributions of the organic matrix would enhance the use of SORS as a rapid, non-invasive diagnostic of bone quality, in vivo and at point-of-care.

### Limitations

A potential limitation to accurate assessment of the lipid and collagen content in the bone samples may be the effect on the organic matrix of alcohol fixation during their storage, which has been found to result in up to 15% bone weight loss compared to unfixed bone^22^. However, the alcohol fixation had no effect on the inorganic matrix^22^. In our study, fixation in alcohol for times ranging between 6–12 months was unavoidable to ensure preservation of the samples for Raman measurements, however, the storage times followed no particular order of age or sex. Rather than assessing the absolute or quantitative measurement of the lipid and collagen content of the samples, in the discussion we have compared the lipid and collagen content between the samples, which we presume would all be similarly affected by the alcohol storage. A thorough study to measure the rate and extent of change to the organic matrix by alcohol should be addressed in future Raman studies.

## Conclusions

Raman spectroscopy measurements can characterize compositional differences in osteoarthritic human cortical bone with regards to age and sex, which is currently not possible with current medical devices optimised for bone analysis. The exquisite sensitivity of the frequencies and relative intensities of molecular vibrations to their structural environment found age-related changes in the collagen and mineral matrix, and differences between older males and females. The complex, overlapping spectral band envelopes for amide I, amide III, CO_3_^2−^ and PO_4_^3−^ vibrational modes were resolved into their individual components that are associated with the secondary structures of the mineral and organic matrices. This allowed more accurate evaluation of the lipid and collagen secondary structure, revealing new information about age- and sex-related changes in the mineral and collagen secondary structure of older (≥ 70 years) and younger (≤ 62 years) males and females.

Younger female and older male bones showed clear differences in lipid and associated secondary collagen structure ratios of the organic matrix, as well as CO_3_^2−^/PO_4_^3−^ ratio. A 2D plot of the Raman band intensities of the 959 cm^−1^ PO_4_^3−^ vs the 1072 cm^−1^ CO_3_^2−^ showed direct proportionality with B-CO_3_^2−^ and PO_4_^3−^ levels spread across a wide range for all four age/sex groups, however, the older females on average presented the lowest levels of B-CO_3_^2−^ and PO_4_^3−^ minerals PCA of fitted band components revealed that older males had the lowest A-CO_3_^2−^ levels, and that older females displayed the greatest increase in B-CO_3_^2−^/PO_4_^3−^ ratio. In addition, older females showed the lowest levels of α-helix structures (1660 cm^−1^ amide 1 and 1271 cm^−1^ amide III modes). These differences can be linked to well-established gender-based changes in physiology.

The Raman technique is non-destructive and very weakly responsive to aqueous environments; with development of spatially offset (SORS) fibre optic probes enabling measurement at cm depths has potential application for in vivo, point-of-care, photonic biomedical diagnostic devices for rapid and non-invasive quantification of the degree of bone mineralization and by proxy, health.

## Supplementary information

Supplementary Information.
