# Supplementary material for: Raman spectroscopy reveals age- and sex-related differences in cortical bone from people with osteoarthritis
Source: Sci Rep. 2020 Nov 10;10:19443. doi: 10.1038/s41598-020-76337-2 (PMC7656243; doi:10.1038/s41598-020-76337-2)
Supplement: Supplementary file 1 — Supplementary Information. [file 41598_2020_76337_MOESM1_ESM.docx]

**Supplementary Information**

**Raman spectroscopy reveals age- and sex-related differences in cortical bone from people with osteoarthritis**

Michel K. Nieuwoudt*^a,b,d,e^, Rayomand Shahlori^a,b,d^, Dorit Naot^c^, Rhea Patel^a,f^, Hannah Holtkamp ^a,b,d^, Claude Aguergaray ^a,e,h^ Maureen Watson^c^, David Musson^c^, Cameron Brown^g^, Nicola Dalbeth^c^, Jillian Cornish^c^, M. Cather Simpson*^a,b,d,e,h^

^a^ The Photon Factory, The University of Auckland, Auckland 1142, New Zealand

^b^ School of Chemical Sciences, the University of Auckland, Auckland, 1142, New Zealand

^c^ Faculty of Medical and Health Sciences, The University of Auckland, Auckland 1142, New Zealand

^d^ The MacDiarmid Institute for Advanced Materials and Nanotechnology, New Zealand

^e^ The Dodd-Walls Centre for Photonic and Quantum Technologies, New Zealand

^f^ Department of Chemical and Materials Engineering, the University of Auckland, Auckland 1142, New Zealand

^g^ Medical Engineering Research Faculty, CPME, IHBI, SEF, Queensland University of Technology, Brisbane, Australia

^h^ Department of Physics, the University of Auckland, Auckland, 1142, New Zealand

***Corresponding authors: [m.nieuwoudt@auckland.ac.nz](mailto:m.nieuwoudt@auckland.ac.nz), [c.simpson@auckland.ac.nz](mailto:c.simpson@auckland.ac.nz)

**Figures**

**
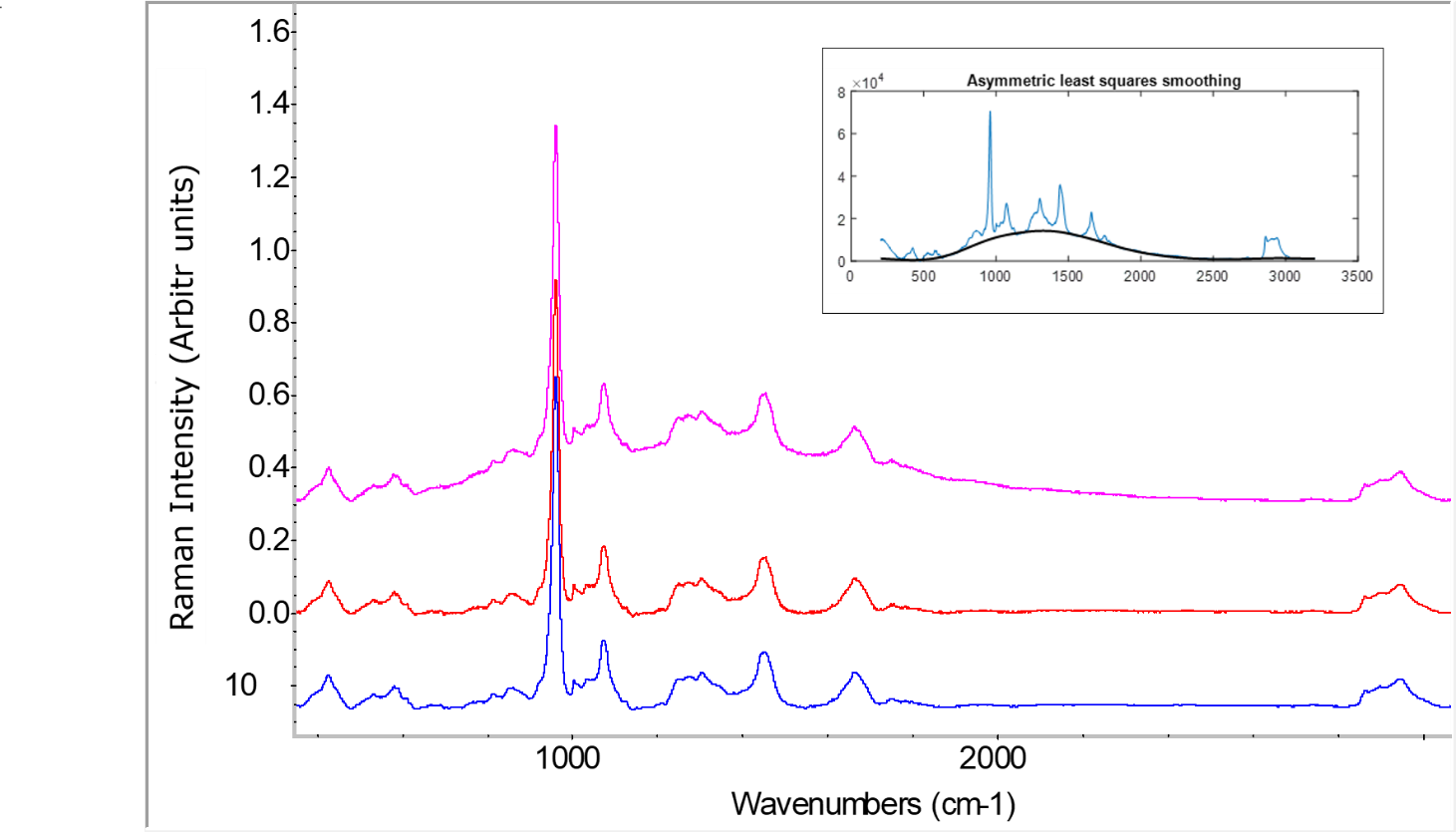
**

**Figure S1**. Baseline corrected (red) and smoothed (blue) spectra of a raw spectrum recorded from one of the bone samples (blue); these were performed using an asymmetric least squares algorithm ^23^ (inset).

**
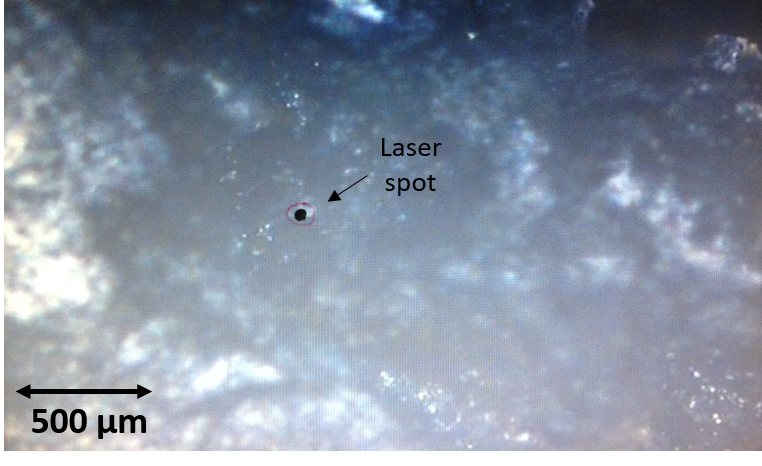
**

**Figure S2** Optical image of the bone cross section of showing heterogeneity of the surface; laser spot size is 1 – 2 µm (size on image not to scale).

**
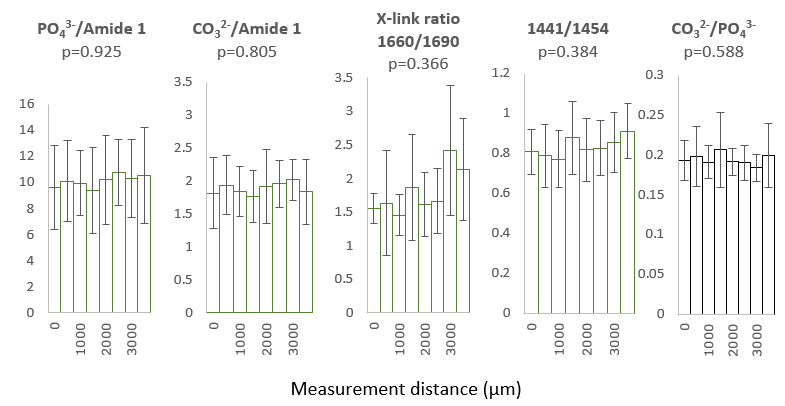
**

**Figure S3**. Average peak height ratios of all samples measured in 500 µm steps, along the surface of the cross section, starting from the periosteal toward the endosteal surface. P values from one way ANOVA of the positions for spectra recorded along the surface are given for each ratio.

**
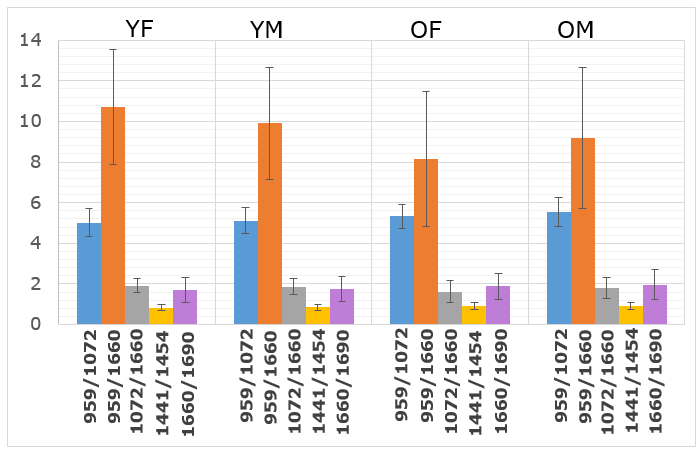
**

**Figure S4.** Band intensities of CO_3_^2-^ and PO_4_^3-^ (relative to the same intensity for the 1450 cm^-1^ –CH_2_ deformation). Data given in Table S3.


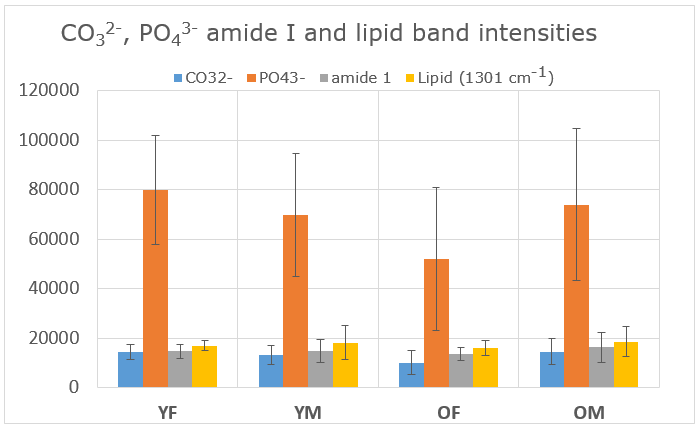


**Figure S5.** Average values for all 158 spectra of the five selected band intensity ratios for the four age/sex groups, relative to the 1450 cm^-1^ –CH_2_ band intensity. (Data given in Table S4).


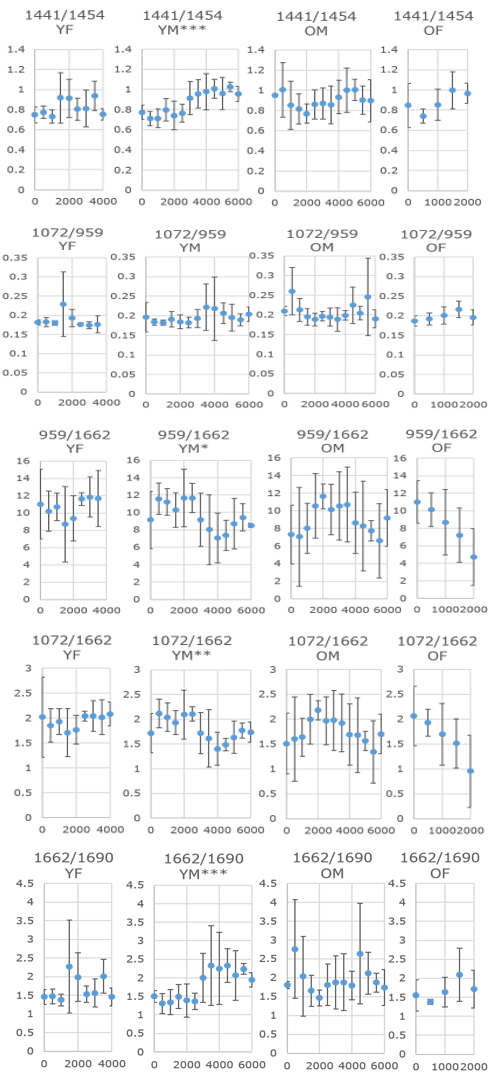


**Figure S6**. Mean values of five selected peak ratios of spectra recorded at 500 um measurement positions from the periosteal to endosteal surface, for each age/sex group. Significant variations with measurement position are labelled, *** p<0.01, ** p<0.05 and *p<0.1 (obtained using a one-way ANOVA).

**Figure S7.** Heat map representing the correlation matrix which shows correlations between the different parameters; these include age, sex, age category, physical parameters (BV/TV, TMD), MM ratio and band intensities of the mineral and collagen components.

#
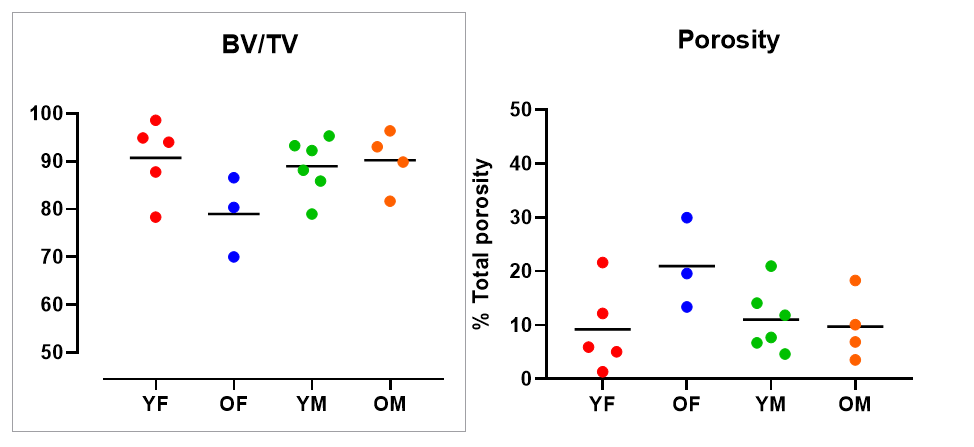


**Figure S8** Scatter plots of the BV/TV and porosity (as 100 – BV/TV) of the 18 samples.

**
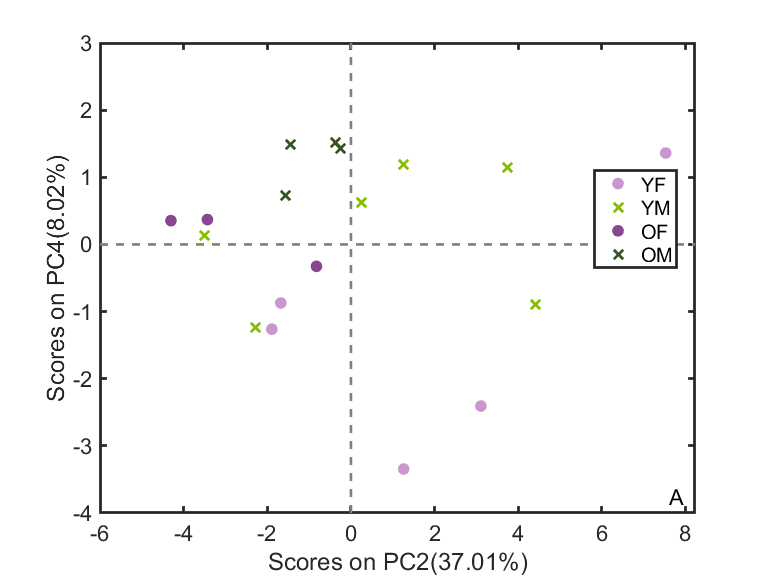
**

**Figure S9(A).** Scores PC1 and PC4 for PCA of the resolved band components in averaged spectra of each of the 18 samples.


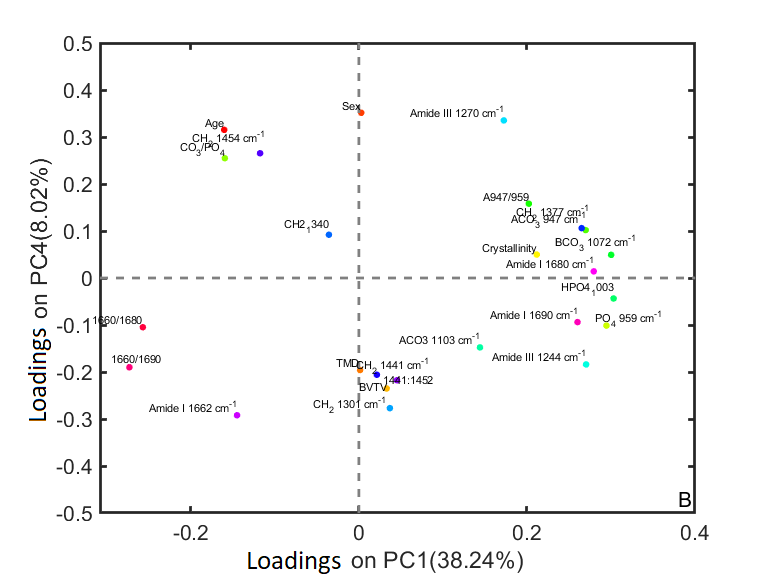


**Figure S9(B).** Loadings PC1 and PC4 for PCA of the resolved band components in averaged spectra of each of the 18 samples.


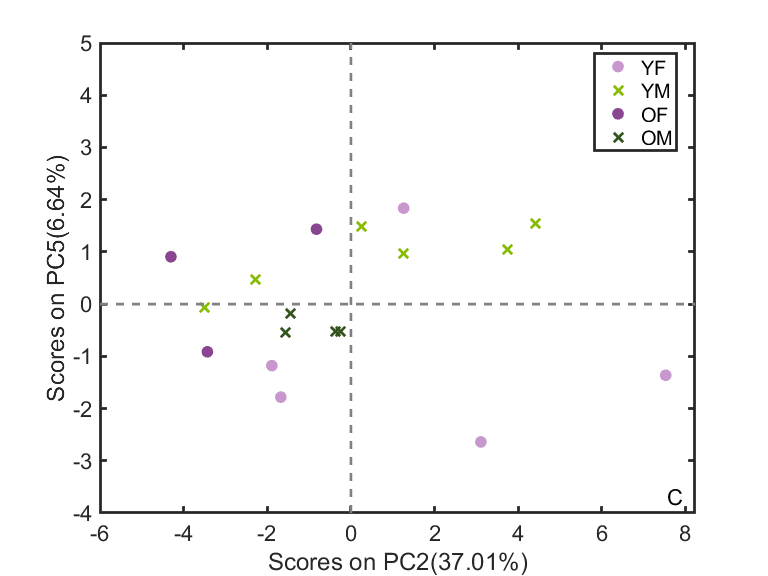


**Figure S9(C).** Scores PC1 and PC5 for PCA of the resolved band components in averaged spectra of each of the 18 samples.


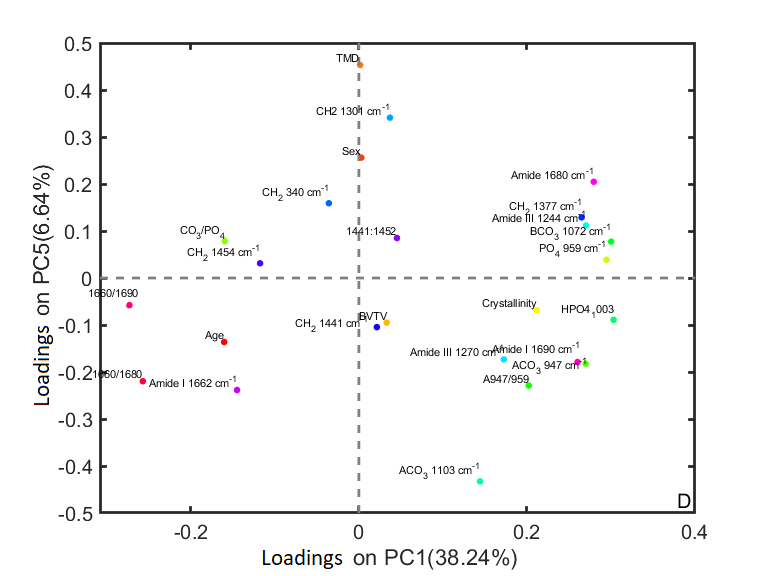


**Figure S9(D).** Loadings PC1 and PC5 for PCA of the resolved band components in averaged spectra of each of the 18 samples.


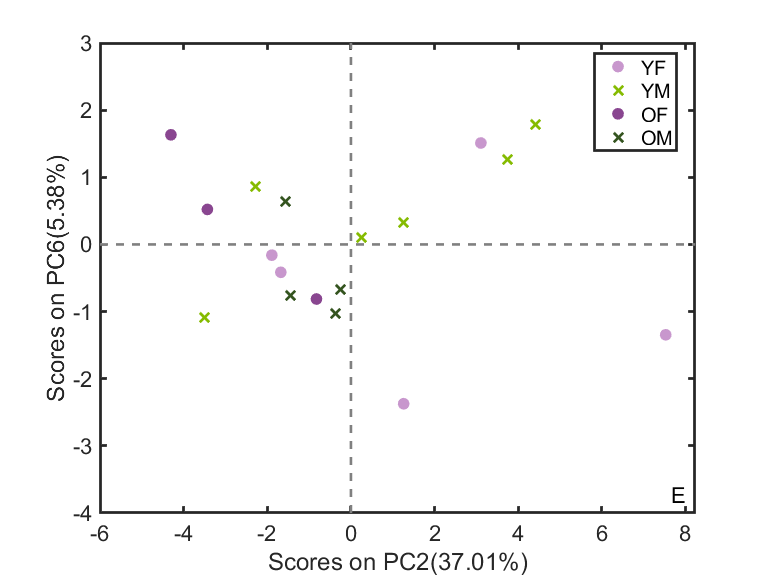


**Figure S9(E).** Scores PC1 and PC6 for PCA of the resolved band components in averaged spectra of each of the 18 samples.

**
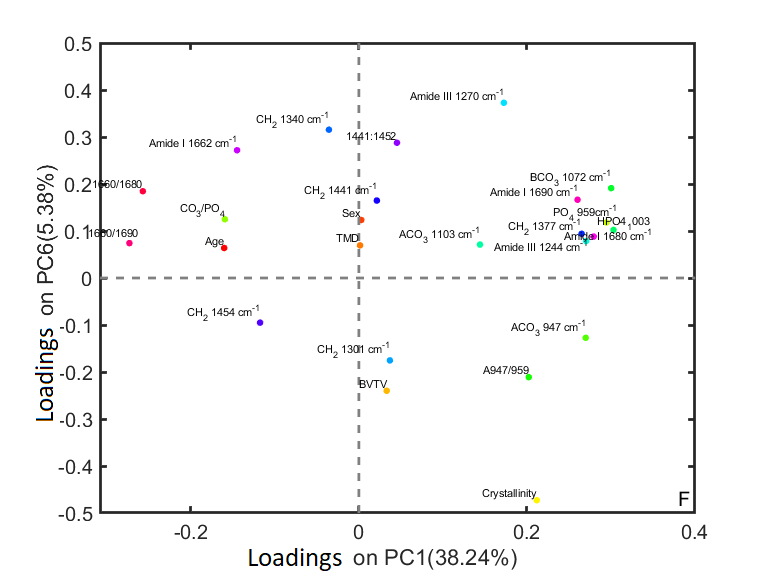
**

**Figure S9(F) L**oadings PC1 and PC6 for PCA of the resolved band components in averaged spectra of each of the 18 samples.

**Tables**

**Table S1.** Bone spectral band height ratios for the 256 combinations contributing to most variation in the spectra. Cells are colour coded according to mineral: mineral ratio (orange), organic: organic ratios (blue) and mineral: organic ratios (grey).

| **Ratio #** | **Peak Ratio** | **Band Ratio** | **Ratio #** | **Peak Ratio** | **Band Ratio** | **Ratio #** | **Peak Ratio** | **Band Ratio** |
| --- | --- | --- | --- | --- | --- | --- | --- | --- |
| R1 | PO_4_^3-^ v_2_/v (S-S) | 428/531 | R71 | Cholesterol/Amide I | 609/1662 | R141 | PO_4_^3-^ v_1_/Amide I | 959/1662 |
| R2 | PO_4_^3-^ v_2_/PO_4_^3-^ v_4_ | 428/582 | R72 | Cholesterol/CH_2_ lipids & proteins | 609/2862 | R142 | PO_4_^3-^ v_1_/CH_2_ lipids & proteins | 959/2862 |
| R3 | PO_4_^3-^ v_2_/Cholesterol | 428/609 | R73 | Cholesterol/C-H stretch | 609/2891 | R143 | PO_4_^3-^ v_1_/C-H stretch | 959/2891 |
| R4 | PO_4_^3-^ v_2_/C-C PO_4_^3-^ v3 ring | 428/859 | R74 | Cholesterol/CH_3_ lipids & proteins | 609/2942 | R144 | PO_4_^3-^ v_1_/CH_3_ lipids & proteins | 959/2942 |
| R5 | PO_4_^3-^ v_2_/C-C stretch, hydroxyproline | 428/868 | R75 | C-C PO_4_^3-^ v3 ring/C-C stretch, hydroxyproline | 859/868 | R145 | HPO_4_^2-^/ PO_4_^3-^ v3 | 1002/1033 |
| R6 | PO_4_^3-^ v_2_/Hydroxyproline | 428/876 | R76 | C-C PO_4_^3-^ v3 ring/Hydroxyproline | 859/876 | R146 | HPO_4_^2-^/CO_3_^2-^ | 1002/1072 |
| R7 | PO_4_^3-^ v_2_/C-C stretch PO_4_^3-^ v3 ring in collagen | 428/920 | R77 | C-C PO_4_^3-^ v3 ring/C-C stretch PO_4_^3-^ v3 ring in collagen | 859/920 | R147 | HPO_4_^2-^/A-CO3 stretch | 1002/1103 |
| R8 | PO_4_^3-^ v_2_/PO_4_^3-^ v_1_ | 428/959 | R78 | C-C PO_4_^3-^ v3 ring/PO_4_^3-^ v_1_ | 859/959 | R148 | HPO_4_^2-^/Amide III | 1002/1246 |
| R9 | PO_4_^3-^ v_2_/HPO42- | 428/1002 | R79 | C-C PO_4_^3-^ v3 ring/HPO42- | 859/1002 | R149 | HPO_4_^2-^/Amide III - v (C-N) | 1002/1273 |
| R10 | PO_4_^3-^ v_2_/ PO_4_^3-^ v3 | 428/1033 | R80 | C-C PO_4_^3-^ v3 ring/ PO_4_^3-^ v3 | 859/1033 | R150 | HPO_4_^2-^/Collagen | 1002/1303 |
| R11 | PO_4_^3-^ v_2_/ CO_3_^2-^ | 428/1072 | R81 | C-C PO_4_^3-^ v3 ring/CO_3_^2-^ | 859/1072 | R151 | HPO_4_^2-^/CH_2_ bending | 1002/1450 |
| R12 | PO_4_^3-^ v_2_/A-CO3 stretch | 428/1103 | R82 | C-C PO_4_^3-^ v3 ring/A-CO3 stretch | 859/1103 | R152 | HPO_4_^2-^/Amide I | 1002/1662 |
| R13 | PO_4_^3-^ v_2_/Amide III | 428/1246 | R83 | C-C PO_4_^3-^ v3 ring/Amide III | 859/1246 | R153 | HPO_4_^2-^/CH_2_ lipids & proteins | 1002/2862 |
| R14 | PO_4_^3-^ v_2_/Amide III - v (C-N) | 428/1273 | R84 | C-C PO_4_^3-^ v3 ring/Amide III - v (C-N) | 859/1273 | R154 | HPO_4_^2-^/C-H stretch | 1002/2891 |
| R15 | PO_4_^3-^ v_2_/Collagen | 428/1303 | R85 | C-C PO_4_^3-^ v3 ring/Collagen | 859/1303 | R155 | HPO_4_^2-^/CH_3_ lipids & proteins | 1002/2942 |
| R16 | PO_4_^3-^ v_2_/CH_2_ bending | 428/1450 | R86 | C-C PO_4_^3-^ v3 ring/CH_2_ bending | 859/1450 | R156 | PO_4_^3-^ v3/CO_3_^2-^ | 1033/1072 |
| R17 | PO_4_^3-^ v_2_/Amide I | 428/1662 | R87 | C-C PO_4_^3-^ v3 ring/Amide I | 859/1662 | R157 | PO_4_^3-^ v3/A-CO3 stretch | 1033/1103 |
| **Ratio #** | **Peak Ratio** | **Band Ratio** | **Ratio #** | **Peak Ratio** | **Band Ratio** | **Ratio #** | **Peak Ratio** | **Band Ratio** |
| R18 | PO_4_^3-^ v_2_/CH_2_ lipids & proteins | 428/2862 | R88 | C-C PO_4_^3-^ v3 ring/CH_2_ lipids & proteins | 859/2862 | R158 | PO_4_^3-^ v3/Amide III | 1033/1246 |
| R19 | PO_4_^3-^ v_2_/C-H stretch | 428/2891 | R89 | C-C PO_4_^3-^ v3 ring/C-H stretch | 859/2891 | R159 | PO_4_^3-^ v3/Amide III - v (C-N) | 1033/1273 |
| R20 | PO_4_^3-^ v_2_/CH_3_ lipids & proteins | 428/2942 | R90 | C-C PO_4_^3-^ v3 ring/CH_3_ lipids & proteins | 859/2942 | R160 | PO_4_^3-^ v3/Collagen | 1033/1303 |
| R21 | v (S-S)/PO_4_^3-^ v_4_ | 531/582 | R91 | C-C stretch, hydroxyproline/Hydroxyproline | 868/876 | R161 | PO_4_^3-^ v3/CH_2_ bending | 1033/1450 |
| R22 | v (S-S)/Cholesterol | 531/609 | R92 | C-C stretch, hydroxyproline/C-C stretch PO_4_^3-^ v3 ring in collagen | 868/920 | R162 | PO_4_^3-^ v3/Amide I | 1033/1662 |
| R23 | v (S-S)/C-C PO_4_^3-^ v3 ring | 531/859 | R93 | C-C stretch, hydroxyproline/PO_4_^3-^ v_1_ | 868/959 | R163 | PO_4_^3-^ v3/CH_2_ lipids & proteins | 1033/2862 |
| R24 | v (S-S)/C-C stretch, hydroxyproline | 531/868 | R94 | C-C stretch, hydroxyproline/ HPO_4_^2-^ | 868/1002 | R164 | PO_4_^3-^ v3/C-H stretch | 1033/2891 |
| R25 | v (S-S)/Hydroxyproline | 531/876 | R95 | C-C stretch, hydroxyproline/ PO_4_^3-^ v3 | 868/1033 | R165 | PO_4_^3-^ v3/CH_3_ lipids & proteins | 1033/2942 |
| R26 | v (S-S)/C-C stretch PO_4_^3-^ v3 ring in collagen | 531/920 | R96 | C-C stretch, hydroxyproline/CO_3_^2-^ | 868/1072 | R166 | CO_3_^2-^/A-CO3 stretch | 1072/1103 |
| R27 | v (S-S)/PO_4_^3-^ v_1_ | 531/959 | R97 | C-C stretch, hydroxyproline/A-CO3 stretch | 868/1103 | R167 | CO_3_^2-^/Amide III | 1072/1246 |
| R28 | v (S-S)/ HPO_4_^2-^ | 531/1002 | R98 | C-C stretch, hydroxyproline/Amide III | 868/1246 | R168 | CO_3_^2-^/Amide III - v (C-N) | 1072/1273 |
| R29 | v (S-S)/ PO_4_^3-^ v3 | 531/1033 | R99 | C-C stretch, hydroxyproline/Amide III - v (C-N) | 868/1273 | R169 | CO_3_^2-^/Collagen | 1072/1303 |
| R30 | v (S-S)/ CO_3_^2-^ | 531/1072 | R100 | C-C stretch, hydroxyproline/Collagen | 868/1303 | R170 | CO_3_^2-^/CH_2_ bending | 1072/1450 |
| R31 | v (S-S)/A-CO3 stretch | 531/1103 | R101 | C-C stretch, hydroxyproline/CH_2_ bending | 868/1450 | R171 | CO_3_^2-^/Amide I | 1072/1662 |
| R32 | v (S-S)/Amide III | 531/1246 | R102 | C-C stretch, hydroxyproline/Amide I | 868/1662 | R172 | CO_3_^2-^/CH_2_ lipids & proteins | 1072/2862 |
| **Ratio #** | **Peak Ratio** | **Band Ratio** | **Ratio #** | **Peak Ratio** | **Band Ratio** | **Ratio #** | **Peak Ratio** | **Band Ratio** |
| R33 | v (S-S)/Amide III - v (C-N) | 531/1273 | R103 | C-C stretch, hydroxyproline/CH_2_ lipids & proteins | 868/2862 | R173 | CO_3_^2-^/C-H stretch | 1072/2891 |
| R34 | v (S-S)/Collagen | 531/1303 | R104 | C-C stretch, hydroxyproline/C-H stretch | 868/2891 | R174 | CO_3_^2-^/CH_3_ lipids & proteins | 1072/2942 |
| R35 | v (S-S)/CH_2_ bending | 531/1450 | R105 | C-C stretch, hydroxyproline/CH_3_ lipids & proteins | 868/2942 | R175 | A-CO3 stretch/Amide III | 1103/1246 |
| R36 | v (S-S)/Amide I | 531/1662 | R106 | Hydroxyproline/C-C stretch PO_4_^3-^ v3 ring in collagen | 876/920 | R176 | A-CO3 stretch/Amide III - v (C-N) | 1103/1273 |
| R37 | v (S-S)/CH_2_ lipids & proteins | 531/2862 | R107 | Hydroxyproline/PO_4_^3-^ v_1_ | 876/959 | R177 | A-CO3 stretch/Collagen | 1103/1303 |
| R38 | v (S-S)/C-H stretch | 531/2891 | R108 | Hydroxyproline/ HPO_4_^2-^ | 876/1002 | R178 | A-CO3 stretch/CH_2_ bending | 1103/1450 |
| R39 | v (S-S)/CH3 lipids & proteins | 531/2942 | R109 | Hydroxyproline/ PO_4_^3-^ v3 | 876/1033 | R179 | A-CO3 stretch/Amide I | 1103/1662 |
| R40 | PO_4_^3-^ v_4_/Cholesterol | 582/609 | R110 | Hydroxyproline/CO_3_^2-^ | 876/1072 | R180 | A-CO3 stretch/CH_2_ lipids & proteins | 1103/2862 |
| R41 | PO_4_^3-^ v_4_/C-C PO_4_^3-^ v3 ring | 582/859 | R111 | Hydroxyproline/A-CO3 stretch | 876/1103 | R181 | A-CO3 stretch/C-H stretch | 1103/2891 |
| R42 | PO_4_^3-^ v_4_/C-C stretch, hydroxyproline | 582/868 | R112 | Hydroxyproline/Amide III | 876/1246 | R182 | A-CO3 stretch/CH_3_ lipids & proteins | 1103/2942 |
| R43 | PO_4_^3-^ v_4_/Hydroxyproline | 582/876 | R113 | Hydroxyproline/Amide III - v (C-N) | 876/1273 | R183 | Amide III/Amide III - v (C-N) | 1246/1273 |
| R44 | PO_4_^3-^ v_4_/C-C stretch PO43- v3 ring in collagen | 582/920 | R114 | Hydroxyproline/Collagen | 876/1303 | R184 | Amide III/Collagen | 1246/1303 |
| R45 | PO_4_^3-^ v_4_/PO_4_^3-^ v_1_ | 582/959 | R115 | Hydroxyproline/CH_2_ bending | 876/1450 | R185 | Amide III/CH_2_ bending | 1246/1450 |
| R46 | PO_4_^3-^ v_4_/ HPO_4_^2-^ | 582/1002 | R116 | Hydroxyproline/Amide I | 876/1662 | R186 | Amide III/Amide I | 1246/1662 |
| R47 | PO_4_^3-^ v_4_/ PO_4_^3-^v3 | 582/1033 | R117 | Hydroxyproline/CH_2_ lipids & proteins | 876/2862 | R187 | Amide III/CH_2_ lipids & proteins | 1246/2862 |
| R48 | PO_4_^3-^ v_4_/CO_3_^2-^ | 582/1072 | R118 | Hydroxyproline/C-H stretch | 876/2891 | R188 | Amide III/C-H stretch | 1246/2891 |
| R49 | PO_4_^3-^ v_4_/A-CO3 stretch | 582/1103 | R119 | Hydroxyproline/CH_3_ lipids & proteins | 876/2942 | R189 | Amide III/CH_3_ lipids & proteins | 1246/2942 |
| R50 | PO_4_^3-^ v_4_/Amide III | 582/1246 | R120 | C-C stretch PO_4_^3-^ v3 ring in collagen/ PO_4_^3-^v_1_ | 920/959 | R190 | Amide III - v (C-N)/Collagen | 1273/1303 |
| **Ratio #** | **Peak Ratio** | **Band Ratio** | **Ratio #** | **Peak Ratio** | **Band Ratio** | **Ratio #** | **Peak Ratio** | **Band Ratio** |
| R51 | PO_4_^3-^ v_4_/Amide III - v (C-N) | 582/1273 | R121 | C-C stretch PO_4_^3-^ v3 ring in collagen/ HPO_4_^2-^ | 920/1002 | R191 | Amide III - v (C-N)/CH_2_ bending | 1273/1450 |
| R52 | PO_4_^3-^ v_4_/Collagen | 582/1303 | R122 | C-C stretch PO_4_^3-^ v3 ring in collagen/ PO_4_^3-^ v3 | 920/1033 | R192 | Amide III - v (C-N)/Amide I | 1273/1662 |
| R53 | PO_4_^3-^ v_4_/CH_2_ bending | 582/1450 | R123 | C-C stretch PO_4_^3-^ v3 ring in collagen/CO_3_^2-^ | 920/1072 | R193 | Amide III - v (C-N)/CH_2_ lipids & proteins | 1273/2862 |
| R54 | PO_4_^3-^ v_4_/Amide I | 582/1662 | R124 | C-C stretch PO_4_^3-^ v3 ring in collagen/A-CO3 stretch | 920/1103 | R194 | Amide III - v (C-N)/C-H stretch | 1273/2891 |
| R55 | PO_4_^3-^ v_4_/CH_2_ lipids & proteins | 582/2862 | R125 | C-C stretch PO_4_^3-^ v3 ring in collagen/Amide III | 920/1246 | R195 | Amide III - v (C-N)/CH_3_ lipids & proteins | 1273/2942 |
| R56 | PO_4_^3-^ v_4_/C-H stretch | 582/2891 | R126 | C-C stretch PO_4_^3-^ v3 ring in collagen/Amide III - v (C-N) | 920/1273 | R196 | Collagen/CH_2_ bending | 1303/1450 |
| R57 | PO_4_^3-^ v_4_/CH_3_ lipids & proteins | 582/2942 | R127 | C-C stretch PO_4_^3-^ v3 ring in collagen/Collagen | 920/1303 | R197 | Collagen/Amide I | 1303/1662 |
| R58 | Cholesterol/C-C PO_4_^3-^ v3 ring | 609/859 | R128 | C-C stretch PO_4_^3-^ v3 ring in collagen/CH_2_ bending | 920/1450 | R198 | Collagen/CH_2_ lipids & proteins | 1303/2862 |
| R59 | Cholesterol/C-C stretch, hydroxyproline | 609/868 | R129 | C-C stretch PO_4_^3-^ v3 ring in collagen/Amide I | 920/1662 | R199 | Collagen/C-H stretch | 1303/2891 |
| R60 | Cholesterol/Hydroxyproline | 609/876 | R130 | C-C stretch PO_4_^3-^ v3 ring in collagen/CH_2_ lipids & proteins | 920/2862 | R200 | Collagen/CH_3_ lipids & proteins | 1303/2942 |
| R61 | Cholesterol/C-C stretch PO_4_^3-^ v3 ring in collagen | 609/920 | R131 | C-C stretch PO_4_^3-^ v3 ring in collagen/C-H stretch | 920/2891 | R201 | CH_2_ bending/Amide I | 1450/1662 |
| R62 | Cholesterol/PO_4_^3-^ v_1_ | 609/959 | R132 | C-C stretch PO_4_^3-^ v3 ring in collagen/CH_3_ lipids & proteins | 920/2942 | R202 | CH_2_ bending/CH_2_ lipids & proteins | 1450/2862 |
| R63 | Cholesterol/ HPO_4_^2-^ | 609/1002 | R133 | PO_4_^3-^ v_1_/ HPO_4_^2-^ | 959/1002 | R203 | CH_2_ bending/C-H stretch | 1450/2891 |
| R64 | Cholesterol/ PO_4_^3-^ v3 | 609/1033 | R134 | PO_4_^3-^ v_1_/ PO_4_^3-^ v3 | 959/1033 | R204 | CH_2_ bending/CH_3_ lipids & proteins | 1450/2942 |
| R65 | Cholesterol/CO_3_^2-^ | 609/1072 | R135 | PO_4_^3-^ v_1_/CO_3_^2-^ | 959/1072 | R205 | Amide I/CH_2_ lipids & proteins | 1662/2862 |
| R66 | Cholesterol/A-CO3 stretch | 609/1103 | R136 | PO_4_^3-^ v_1_/A-CO3 stretch | 959/1103 | R206 | Amide I/C-H stretch | 1662/2891 |
| **Ratio #** | **Peak Ratio** | **Band Ratio** | **Ratio #** | **Peak Ratio** | **Band Ratio** | **Ratio #** | **Peak Ratio** | **Band Ratio** |
| R67 | Cholesterol/Amide III | 609/1246 | R137 | PO_4_^3-^ v_1_/Amide III | 959/1246 | R207 | Amide I/CH_3_ lipids & proteins | 1662/2942 |
| R68 | Cholesterol/Amide III - v (C-N) | 609/1273 | R138 | PO_4_^3-^ v_1_/Amide III - v (C-N) | 959/1273 | R208 | CH_2_ lipids & proteins/C-H stretch | 2862/2891 |
| R69 | Cholesterol/ Collagen | 609/1303 | R139 | PO_4_^3-^ v_1_/Collagen | 959/1303 | R209 | CH_2_ lipids & proteins/CH_3_ lipids & proteins | 2862/2942 |
| R70 | Cholesterol/CH_2_ bending | 609/1450 | R140 | PO_4_^3-^ v_1_/CH_2_ bending | 959/1450 | R210 | C-H stretch/CH_3_ lipids & proteins | 2891/2942 |

**Table S2:** Bands in curve fitted spectra / used to obtain the ratios in Table 2, with assignments and references.

| Band | Band assignment | References |
| --- | --- | --- |
| 424, 432, 426 cm^-1^ | PO_4_^3-^ v_2_ :asymmetric stretch | ^1,2^ |
| 447, 452 cm^-1^ | PO_4_^3-^ v_2_ :asymmetric stretch, Hydroxyproline | ^1,2^ |
| 531 cm^-1^ | PO_4_^3-^ v_4_out of plane deformation | ^3^ |
| 581, 584, 590, 611 cm^-1^ | PO_4_^3-^ v_4_ (P-O) asymmetric deformation | ^1,2^ |
| 859, 868 cm^-1^ | Proline, hydroxyproline and collagen backbone ν_s_(C-C), δ(C-CH) and CH_3_ν_s_(C-H) | ^4^ |
| 873 cm^-1^ | P-OH stretch | ^2^ |
| 876, 920 cm^-1^ | Proline, hydroxyproline and collagen backbone ν_s_(C-C), δ(C-C), δ(C-C), δ(C=O), CH_3_ν_s_(C-H), CH_2_δ(C-H), confined H_2_O δ (O-H) & ν_s_(O-H), δ(C=O) | ^4^ |
| 947, 947 cm^-1^ | A-type CO32- substitution in bioapatite | ^1^ |
| 956, 959, 961, 1044 cm^-1^ | PO_4_^3-^ v_1_  (P-O) symmetric stretch | ^1,2,5^ |
| 1002, 1005 cm^-1^ | HPO_4_^2-^ **ν_1_**(O-P-O), phenyalanine ν_s_(C-C), CH_3_ν_s_(C-H), CH_2_δ(C-H), CH_3_ν_s_(C-H) & CH_3_ν_as_(C-H) | ^2,4,6,7^ |
| 1032, 1033, 1034 cm^-1^ | PO_4_^3-^ v_3_ δ_as_(O-P-O) asymmetric deformation, proline | ^1,2,8^ |
| 1047 cm^-1^ | PO_4_^3-^ v_3_ δ(O-P-O), C-N stretching | ^1^ |
| 1059 cm^-1^ | ν(C-C)*_trans_*, phospholipids | ^1^ |
| 1070, 1071, 1072,  1077 cm^-1^ | B - substituted CO_3_^2-^, ν_s_(C-O)  HPO_4_^2-^ and PO_4_^3-^ stretching | ^1,2,6,8,9^ |
| 1103 cm^-1^ | A - substituted CO_3_^2-^ ν_s_(C-O)  Collagen, amide III ν(C-C), ν(C-O), ν (C-N), δ(C=O), CH_3_ν_s_(C-H), CH_2_δ(C-H), CH_3_ν_s_(C-H), NH_2_ν_s_(N-H), CH_2_δ(C-H), CH_3_δ(C-H) | ^2,4,6^ |
| 1243, 1246 cm^-1^ | β-sheets and random coils amide III,  C-N stretching and C-H deformation and CH_3_ symmetrical stretching | ^2,4,9-11,1^ |
| 1271, 1273 cm^-1^ | α-helices amide III  C-N stretching and C-H deformation and CH_3_ symmetrical stretching | ^9,11,1,4,10^ |
| 1300,1303 cm^-1^ | δ(=CH) phospholipids, δ_as_(CH_3_) and δ(CH_2_) deformation collagen and lipids | ^1,4,6,12,13^ |
| 1323 cm^-1^ | α-fibrous AmIII C-N stretching and C-H deformation and CH_3_ symmetrical stretching | ^1^ |
| 1342 cm^-1^ | α-helices amide III  C-N stretching and C-H deformation and CH_3_ symmetrical stretching | ^1^ |
| 1439, 1449,1451 cm^-1^ | δ_as_(CH_3_) and δ(CH_2_) deformation of proteins, NH_2_ stretching and deformation of collagen, lipids | ^1,2,4,10,14^ |
| 1465, 1478 | Fermi interaction δ(CH_2_) and γ(CH_2_) | ^1^ |
| 1624, 1631, 1637, 1645 | β-sheets and random coils, turns and bends - amide I C=C and C=O stretching, NH_2_ stretching and deformation | ^1,15^ |
| 1654 cm^-1^, 1660, 1670, 1675, 1684, cm^-1^ | α-helices - amide I C=C and C=O stretching, NH_2_ stretching and deformation | ^15^ |
| 1660-1662 cm^-1^ | Pyridinoline, Amide I C=C and C=O stretching in collagen**,** CH2/CH_3_ stretching and deformation, NH_2_ stretching and deformation of lipids & collagen | ^1,2,4,16^ |
| 1690 cm^-1^ | β-sheets and random coils - amide I C=C and C=O stretching, NH_2_ stretching and deformation | ^4,8,11,15^ |
| 2852, 2856, 2862, 2882, 2886 cm^-1^ | νCH_2_ lipids, C-H stretching | ^1,2,17,18^ |
| 2975, 2890, 2891 cm^-1^ | ν_sym_CH_2_ lipids, collagen | ^1,2,10^ |
| 2920 cm^-1^ | ν_asym_CH_2,_ polypeptides | ^1^ |
| 2936, 2938, 2942, 2946, 2950 cm^-1^, | ν_asym_CH_3,_ proteins and lipids | ^2,10,17^ |
| 2972, 2975,2978, 2  986 cm^-1^ | C-H stretching, collagen | ^1,2^ |
| 3001 cm^-1^ | ν(–C=C–H) stretching | ^1^ |

**Table S3.** Averaged band intensity ratios of all spectra, and microCT measurements for the four age/sex groups

| Group | 959/1072 | 959/1660 | 1072/1660 | 1441/1454 | 1660/1690 | TMD | Porosity |
| --- | --- | --- | --- | --- | --- | --- | --- |
| YF | 5.01 | 10.72 | 1.92 | 0.83 | 1.71 | 1.09 | 9.24 |
| YM | 5.12 | 9.91 | 1.85 | 0.84 | 1.74 | 1.09 | 11.00 |
| OF | 5.32 | 8.16 | 1.61 | 0.91 | 1.87 | 1.10 | 20.99 |
| OM | 5.55 | 9.18 | 1.79 | 0.89 | 1.96 | 1.09 | 9.73 |

**Table S4** Averaged band intensities (from all spectra) of CO_3_^2-^, PO_4_^3-^, 1662 cm^-1^ amide I and 1301 cm^-1^ lipid bands for the four age/sex groups.

| Group | CO_3_^2-^ | PO_4_^3-^ | amide 1 | Lipid (1301 cm^-1^) |
| --- | --- | --- | --- | --- |
| YF | 14397.75 | 79883.40 | 14651.89 | 17003.21 |
| YM | 13074.63 | 69615.95 | 14782.89 | 18238.32 |
| OF | 10180.62 | 52075.78 | 13608.71 | 15974.36 |
| OM | 14539.05 | 73948.87 | 16381.92 | 18659.19 |

**REFERENCES**

1 Dougherty, G. Quantitative CT in the measurement of bone quality Quantitative CT in the measurement of bone quantity and bone quality for assessing osteoporosis *Med. Eng. Phys* **18**, 557-568 (1996).

2 Boskey, A. L. & Coleman, R. Aging and bone. *J Dent Res* **89**, 1333-1348 (2010).

3 Timlin, J.A., Carden, A. & Morris, M.D. Chemical microstructure of cortical bone probed by Raman transects. *Appl. Spectrosc.* **53**, 1429-1435 (1999).

4 Nair, A. K., Gautieri, A., Chang, S. W. & Buehler, M. J. Molecular mechanics of mineralized collagen fibrils in bone. *Nat Commun* **4**, 1724, (2013).

5 Grynpas MD, Tupy. J.H & Sodek J. The distribution of soluble, mineralbound, and matrix-bound proteins in osteoporotic and normal bones. *Bone* **15**, 505-513 (1994).

6 Legros, R., Balmain, N. & Bonel, G.. Age-Related Changes in Mineral of Rat and Bovine Cortical Bone. *Calcif Tissue Int* **41**, 137-144 ( 1987).

7 Duran, I., Martakis, K., Hamacher, S., Stark, C., Semler, O. & Schoenau, E*.* Are there effects of age, gender, height, and body fat on the functional muscle-bone unit in children and adults? *Osteoporosis International* **29**, 1069-1079 (2018).

8 France, C. A. M., Thomas, D. B., Doney, C. R. & Madden, O. FT-Raman spectroscopy as a method for screening collagen diagenesis in bone. *Journal of Archaeological Science* **42**, 346-355 (2014).

9 Singh, L., Tyagi, S., Myers, D. & Duque, G. Good, bad, or ugly: the biological roles of bone marrow fat. *Current Osteoporosis Reports* **16**, 130-137 (2018).

10 Carden, A. & Morris, M. D. Application of vibrational spectroscopy to the study of mineralized tissues (review). *Journal of Biomedical Optics* **5**, 259-268 (2000)11 Mandair, G. S. & Morris, M. D. Contributions of Raman spectroscopy to the understanding of bone strength. *Bonekey Reports* 4, doi:10.1038/bonekey.2014.115 (2015).

12 Pavicevic, A., Glumac, S., Sopta, J., Popovic-Bijelic, A., Mojovic, M. & Bacuc, G. Raman microspectroscopy as a biomarking tool for in vitro diagnosis of cancer: a feasibility study. *Croatian Medical Journal* **53**, 551-557 (2012).

13 Unal, M. & Akkus, O. Raman spectral classification of mineral- and collagen-bound water's associations to elastic and post-yield mechanical properties of cortical bone. *Bone* **81**, 315-326 (2015).

14 Morris, M.D. & Mandair, G.S. Raman assessment of bone quality. *Clin Orthop Relat Res* **469**, 2160-2169 (2011).

15 Chan GK, Duque, G. Age-related bone loss: old bone, new facts. *Gerontology* **48**, 62-71 (2002).

16 Zioupos, P., Currey, J.D. & Hamer, A.J. The role of collagen in the declining mechanical propertiesof aging human cortical bone. *J Biomed Mater Res.* **45**, 108-116 (1999).

17 Melton L.J. in *Osteoporosis: Etiology, diagnosis and management* (ed Melton L.J. Riggs B.L.) 133-154 (Raven Press, New York, 1988).

18 Yates, L.B., Karasik, D., Beck, T.J., Cupples, L.A. & Kiel, D.P. Hip structural geometry in old and old-old age: similarities and differences between men and women. *Bone* **41**, 722-732 (2007).

19 Donnelly, E., Boskey, A. L., Baker, S. P. & van der Meulen, M. C. Effects of tissue age on bone tissue material composition and nanomechanical properties in the rat cortex. *J Biomed Mater Res A* **92**, 1048-1056 (2010).

20 Paschalis, E. P., Gamsjaeger, S., Hassler, N., Klaushofer, K. & Burr, D. Ovarian hormone depletion affects cortical bone quality differently on different skeletal envelopes. *Bone* **95**, 55-64 (2017).

21 Paschalis, E. P. Fratzl, P., Gamsjaeger, S., Hassler, N., Brozek, W., Eriksen, E. F., Rauch, F., Glorieux, F. H., Shane, E., Dempster, D., Cohen, A., Recker, R., Klaushofer, K., Aging Versus Postmenopausal Osteoporosis: Bone Composition and Maturation Kinetics at Actively-Forming Trabecular Surfaces of Female Subjects Aged 1 to 84 Years. *J Bone Miner Res* **31**, 347-357 (2016).

22 de Rooi, J. J. & Eilers, P. H. C. Mixture models for baseline estimation. *Chemometrics and Intelligent Laboratory Systems* **117**, 56-60 (2012).

23 Eilers, P. A Perfect Smoother. *Analytical Chemistry* **75**, 3299-3304 (2003).

24 Khalid, M., Bora, T., Ghaithi, A. A., Thukral, S. & Dutta, J. Raman Spectroscopy detects changes in Bone Mineral Quality and Collagen Cross-linkage in Staphylococcus Infected Human Bone. *Sci Rep* **8**, 9417 (2018).

25 Penel, G., Leroy, G. Rey, C. & Bres, E. MicroRaman spectral study of the PO_4_ and CO_3_ vibrational modes. *Calcif Tissue Int* **63,** 475-481 (1998).

26 Awonusi, A., Morris, M. D. & Tecklenburg, M. M. Carbonate assignment and calibration in the Raman spectrum of apatite. *Calcif Tissue Int* **81**, 46-52, (2007).

27 Nguyen, T.T., Gobinet, C., Feru, J., Pasco, S.B., Manfait, M. & Piot, O*.* Characterization of type I and IV collagens by Raman microspectroscopy: identification of spectral markers of the dermo-epidermal junction. *Spectroscopy: An International Journal* **27**, 421-427 (2012).

28 Lakshmi, R.J., Alexander, M., Kurien, J., Mahato K.K. & Kartha, V.B. Osteoradionecrosis (ORN) of the mandible: a laser Raman spectroscopic study. *Appl. Spectrosc.* **57**, 1100-1116 (2003).

29 Sato, E. T. & Martinho, H. First-principles calculations of Raman vibrational modes in the fingerprint region for connective tissue. *Biomed Opt Express* **9**, 1728-1734 (2018).

30 Farley, D., Duclos, M.E., Gineyts, E., Bertholon, C., Viguet-Carrin, S., Nallala, J., Sockalingum, G.D., Bertrand, D., Roger, T., Hartmann, D.J., Chapurlat, R., Boivin & G. The ratio 1660/1690 cm^-1^ measured by IR microspectroscopy is not specific of enzymatic collagen cross-linking in bone tissue. *PLoSOne* **6**, e28736 (2011).

31 Fowler, B.O., Markovic, M., Brown, W.E. Octocalcium phosphate. 3. Infrared and Raman vibrational spectra. *Chemical Materials* **5**, 1417-1423 (1993).

32 Movasaghi, Z., Rehman, S. & Rehman, I. U. Raman spectroscopy of biological tissues. *Appl. Spectrosc. Rev.* **42**, 493-541 (2007).

33 Paschalis, E.P., Verdelis, K. Doty, S.B., Boskey, A.L. & Mendelsohn, R. Spectroscopic characterizarion of collagen cross-links in bone. *J. Bone Miner Res* **16**, 1821-1828 (2001).

34 Schrof, S., Varga, P., Hesse, B., Schone, M., Schutz, R., Masic, A., Raum, K., Multimodal correlative investigation of the interplaying micro-architecture, chemical composition and mechanical properties of human cortical bone tissue reveals predominant role of fibrillar organization in determining microelastic tissue properties. *Acta Biomater* **44**, 51-64, (2016).

35 McNerny, E. M., Gong, B., Morris, M. D. & Kohn, D. H. Bone fracture toughness and strength correlate with collagen cross-link maturity in a dose-controlled lathyrism mouse model. *J Bone Miner Res* **30**, 455-464 (2015).

36 Pallu, S., Chappard, C., Kewish, C. M., Jaffré, C. & Portier, H. New insights in osteocyte imaging by synchrotron radiation. *Journal of Spectral Imaging*, **9**, a3 (2020).

37 Gamsjaeger, S., Robins, S.P., Tatakis, D.N., Klaushofer, K. & Paschalis, E.P. Identification of pyridinoline trivalent collagen cross-links by Raman microspectroscopy. *Calcified Tissue International* **100**, 565–574 (2017).

38 Goldberg M. & Boskey. A.L., Lipids and biomineralizations. *Prog Histochem Cytochem.* **31**, 1-187 (1996).

39 Boskey A.L & Reddi, A.H., Changes in lipids during matrix: induced endochondral bone formation. *Calcif Tissue Int.* **35**, 549 – 554 (1983).

40 Rey, C., Collins, B., Goehl, T., Dickson,I.R., & Glimcher, M.. The Carbonate Environment in Bone Mineral: A Resolution-Enhanced Fourier Transform Infrared Spectroscopy Study *Calcif Tissue Int* **45**, 157-164 (1989).

41 Bala Y, Farlay, D. & Boivin G., Bone mineralization: from tissue to crystal in normal and pathological contexts. *Osteoporos Int.* **24**, 2153–2166 (2013).

42 Farlay, D., Duclos, M-E., Gineyts, E., Bertholon, C., Viguet-Carrin, S., Nallala, J., Sockalingum, G.D., Bertrand, Roger, T., Hartmann, D., Chapurlat, R. & Bolvin, G. The ratio 1660/1690 cm^-1^ measured by infrared microspectroscopy is not specific of enzymatic collagen cross-links in bone tissue. *PLoS One* **6**, e28736 (2011).

43 Penel, G., Delfosse, C., Descamps, M. & Leroy, G. Composition of bone and apatitic biomaterials as revealed by intravital Raman microspectroscopy. *Bone* **36**, 893-901 (2005).

44 Gaber, B.P., Yager, P & Peticolas, W.L. Conformational nonequivalence of chains 1 and 2 of dipalmitoyl phosphatidylcholine as observed by Raman spectroscopy.  *Biophysics Journal* **24**, 677-688 (1978).

45 Styner, M., Thompsons, W.R., Galior, K., Uzer, G., Wu, X., Kadari, S., Case, N., Zie, Z., Sen, B., Romaine, A., Pagnotti, G.M., Rubin, C.T., Styner, M.A., Horowitz, M.C. & Rubin, J. Bone marrow fat accumulation accelerated by high fat diet is suppressed by exercise. *Bone* **64**, 39-46 (2014).

46 During, A., Penel, G. & Hardouin, P. Understanding the local actions of lipids in bone physiology. *Prog Lipid Res* **59**, 126-146 (2015)

47 Bouxsein, M.L., Boyd, S.K., Christiansen, B.A., Guldberg, R.E., Jepsen, K.J. & Muller, R. Guidelines for assessment of bone microstructure in rodents using micro-computed tomography. *J Bone Miner Res* **25**, 1468-1486 (2010).

48 Morgan, E.F., Unnikrisnan, G.U. & Hussein, A.I.. Bone Mechanical Properties in Healthy and Diseased States. *Annual Review of Biomedical Engineering* **20,** 119-143 (2018).

49 Unal, M., Creecy, A. & Nyman, J. S. The Role of Matrix Composition in the Mechanical Behavior of Bone. *Curr Osteoporos Rep* **16**, 205-215 (2018).

50 Akkus, O., Adar, F. & Schaffler, M. B. Age-related changes in physicochemical properties of mineral crystals are related to impaired mechanical function of cortical bone. *Bone* **34**, 443-453 (2004).

51 Gautieri, A. R., Buehler, M.J., Vesentini., S. Age- and diabetes-related nonenzymatic crosslinks in collagen fibrils: candidate amino acids involved in AGEs. *Matrix Biology* **34**, 89-95 (2014).

52 Kotha, S.P. & Guzelsu, N. Effect of bone mineral content on the tensile properties of cortical bone: Experiments and theory. *J Bio-mech Eng: Trans ASME* **125**, 785-793 (2004).

53 Rahagavan, M., Sahar, N.D., Kohn, D.H. & Morris, M.D. Age-specific profiles of tissue-level composition and mechanical propertie of murine cortical bone. *Bone* **50**, 942-953 (2012).

54 Paschalis, E. P., Gamsjaeger, S., Tatakis, D. N., Hassler, N., Robins, S. P. & Klaushofer, K., Fourier transform Infrared spectroscopic characterization of mineralizing type I collagen enzymatic trivalent cross-links. *Calcif Tissue Int* **96**, 18-29 (2015).

55 Ozaki, Y. Medical applications of Raman spectroscopy, *Appl. Spectrosc. Rev.* **24**, 259-312 (1988).
